# A synthetic pseudopotential benchmark for testing production pseudopotentials and constructing pseudopotentials with chemical accuracy

**DOI:** 10.1039/d6cp00528d

**Published:** 2026-09-08

**Authors:** Andris Gulans, Anders Brakestad, Stig Rune Jensen, Moritz Gubler, Luca Frediani, Stefan Goedecker

**Affiliations:** a Faculty of Science and Technology, University of Latvia Jelgavas iela 3 LV-1003 Riga Latvia andris.gulans@lu.lv; b Department of Physics, Universitaet Basel Klingelbergstrasse 82 CH-4056 Basel Switzerland stefan.goedecker@unibas.ch; c Hylleraas Centre for Quantum Molecular Sciences, UiT The Arctic University of Norway 9037 Tromsø Norway; d Department of Chemistry, UiT The Arctic University of Norway 9037 Tromsø Norway; e PSI Center for Scientific Computing, Theory and Data, Paul Scherrer Institute CH-5232 Villigen PSI Switzerland

## Abstract

Norm-conserving pseudopotentials guarantee that the electrostatic monopoles arising from the all-electron and pseudo wavefunctions are identical. We introduce a generalization of this concept, that also guarantees that higher multipoles arising from a hybridization of the orbitals are approximately conserved. By including in addition shallow core states we can achieve errors in the atomization energies within density functional theory that are always smaller than the so-called chemical accuracy of 1 kcal mol^−1^. To demonstrate the accuracy of our pseudopotentials as well as to assess the accuracy of other pseudopotentials we generated a synthetic benchmark of atomization energies in highly precise all-electron calculations. The data bank contains 669 molecules with 65 chemical elements from the first six periods of the periodic table. In contrast to other databases, ours is designed for calculations with systematic basis sets such as plane waves or wavelets. At the same time our molecular test set contains a much larger structural variety than test sets based on periodic bulk systems. Based on our highly accurate multipole conserving pseudopotentials we introduce a scheme that allows us to disentangle the errors in atomization energies by assigning the energy error contributions from the pseudopotentials of the different elements in a molecule to the individual pseudopotentials of these elements. In this way low quality pseudopotentials for certain elements can easily be detected. Using this approach we tested the default pseudopotentials provided by major codes such as ABINIT or QE and find that the errors for some elements are quite large and are in many cases actually larger than the errors arising from the PBE exchange correlation functional.

## Abstract 2

We use a 669-molecule benchmark set with highly accurate all-electron atomization energies and multipole-conserving pseudopotentials to perform an element-resolved assessment of production pseudopotentials.

## Graphical abstract

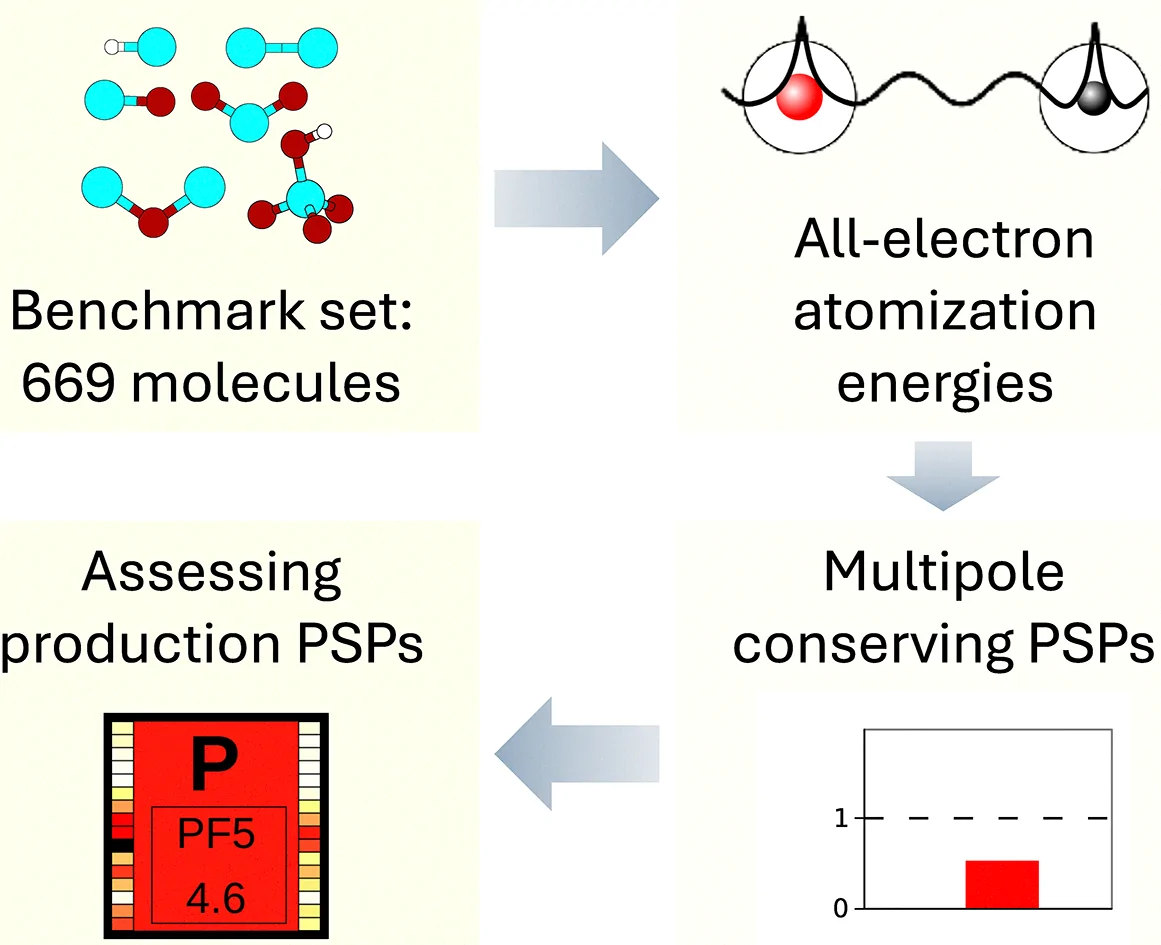

Modern computational materials research relies heavily on density-functional theory (DFT), and a lot of computational effort is dedicated to the design and discovery of novel materials. This type of research revolves around high-throughput calculations and accumulation of data for further processing. There are initiatives that create and maintain multi-purpose databases with millions of individual DFT calculations. The data are used for material screening *via* direct inspection of modeled properties or predictions based on machine-learning algorithms that use the accumulated DFT results as an input. This database-driven approach has already led to finding promising materials for high-strength corrosion-resistant steel,^1^ thermoelectrics,^2^ magnetocalorics,^3^ and beyond.

A large fraction of DFT calculations rely on pseudopotentials (PSPs) – a mathematical construct that eliminates core electrons and simplifies the description of valence electrons by replacing rapidly varying wave functions by smooth valence pseudo-wavefunctions. This approach makes electronic structure calculations so robust and efficient that it has become a common choice for high-throughput studies, and such prominent data projects as Materials Project,^4^ AflowLib,^5^ OQMD^6^ and the perovskite database^7^ rely exclusively on PSPs.

With billions of CPU hours spent in DFT calculations with PSPs yearly, it is highly important to make sure that they are reliable. In particular, predictive computational chemistry requires so-called chemical accuracy (1 kcal mol^−1^) in energies. Although the quality of PSPs is gradually evolving,^8,9^ they introduce numerical errors that cannot be contained within this limit.

The quality of PSPs is commonly assessed in benchmark calculations by comparison with numerically precise all-electron reference data. At present, there are three data sets that are actively used for this purpose.^8–10^ All of them contain only solid-state systems, both elemental and multi-component (oxides and perovskites), and provide reference data from all-electron full-potential calculations employing linearized augmented waves (LAPW) plus local orbitals (LO).^11–13^ The quantities chosen for the comparison with the LAPW+LO reference were based on the equation of state, namely, the lattice constants, the bulk modulus, the *Δ*-measure and further elaborate metrics defined in ref. 14 and 9. Despite being difficult to interpret, the *Δ*-measure in particular has become the *de facto* target for optimizing the PSP parameters. Considering the diversity of systems and properties studied in real materials applications, it is clear that the currently available benchmark sets do not provide a sufficient insight into the PSP performance.

In this paper, we introduce a new benchmark set aiming at chemical diversity beyond what is currently available and obtain highly converged all-electron atomization energies within the PBE parametrization of the exchange–correlation functional.^15^ We employ specifically PBE, because this is the most commonly used functional in materials science and the aforementioned data projects. We also introduce new PSPs designed to conserve not only the norm of the charge, but also reproduce the multipole moments arising from chemically relevant hybridizations. Our extensive validation shows that chemical accuracy (with respect to all-electron reference) is guaranteed with these PSPs. We also employ them for decomposing pseudization errors in heteronuclear systems into individual atomic contributions. This approach is used for assessing transferability of commonly used open-source PSP families generated using the PBE exchange–correlation functional: GBRV,^10^ ONCV,^16,17^ HGH,^18^ kjpaw, rrkjus,^19^ JTH,^20^ SSSP (precision and efficiency)^21^ available in electronic-structure packages such as quantum espresso (QE).^22^ We extend the analysis also to the projector-augmented-wave (PAW) data sets^23,24^ used in the VASP code.^25,26^ Our benchmark is a synthetic one which means that the molecular structures in this set are not real physical systems. Instead, they are chosen such that the calculations required to validate a new PSP can be done without much compute time and without much human intervention. Nevertheless, validation results obtained from this benchmark are highly transferable to real physical systems.

## 1 Multipole-conserving pseudopotentials

Modern high-throughput studies require a high degree of transferability of PSPs, as this type of calculations consider chemically diverse materials that should be described with consistent precision. Chemical diversity, in turn, means that a given atomic species can be placed in different environments potentially resulting in wide range of ionic charges and multipole moments. Let us recall that the multipole *Q*_*ℓm*_ of a charge distribution *ρ*(**r**) is defined as.1
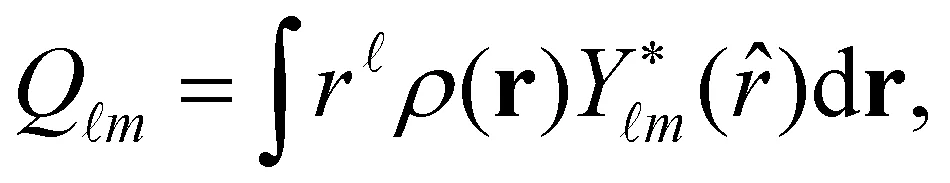
where the argument of the spherical harmonics *Y*_*ℓm*_(*r̂*) is a unit vector. From the various electronic-structure methods using muffin-tins (MT), we know that within an MT the wavefunctions can be written as a sum of a small number of wavefunctions that are products of radial functions *R*_*nℓ*_(*r*) and spherical harmonics *Y*_*ℓm*_(*r̂*). *R*_*nℓ*_(*r*) comprise atomic-like functions plus a few additional ones typically with energy derivatives that allow us to obtain the complete-basis limit efficiently. The resulting charge density contains then cross terms between different *n*, *ℓ* and *m* and their multipoles are given by2
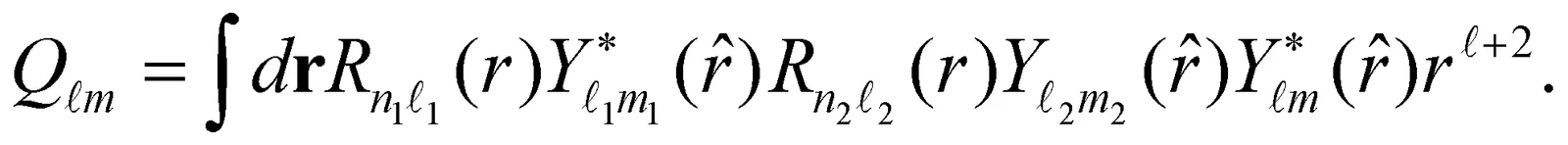


While norm conservation^27^ ensures that the monopoles match, higher multipoles do not. The higher multipoles arising from the charge distribution in the spheres of different atoms interact and give thus a contribution to the atomization energy. In order to describe correctly this interaction of all relevant multipoles, we introduce the concept of multipole conservation. A PSP with the property that the *Q*_*ℓm*_'s of eqn (2) are identical if either calculated with the all-electron or the pseudo wavefunctions will be called multipole conserving (MPC) PSP. It is thus a generalization of the norm conservation property where only the monopoles have to be identical. In practice, the relation has to be fulfilled of course only for those orbitals with quantum numbers *n* and *ℓ* that actively participate in chemical bonding. Such a property can be enforced since, in principle, the pseudo wavefunction within the sphere of is arbitrary. While introducing constraints of this type would be highly problematic if the PSP is constructed by an inversion of the radial Schrödinger equation,^28^ they can easily be handled if the PSP is constructed by a fitting procedure.^29^ As analytical form, we use the dual-space Gaussian-type PSP since it allows for multiple projectors and has optimality properties with respect to a real space implementation.^29^ The construction of the new MPC PSPs is performed following the methodology described in ref. 30 while fitting the following quantities:

• The Kohn–Sham (KS) valence eigenvalues of the pseudo-atom for the atomic ground state as well as for several excited and ionized configurations within several confinement potentials have to agree to within 1 mHa or better with the all-electron eigenvalues. The eigenvalues of semi-core states are fitted with lower accuracy than those of the valence states. For very deep semicore states (about −10 Ha) the precision can be lowered up to 100 mHa. The monopoles have to agree to within 10^−3^ or better since they represent the important norm conservation condition.

• Higher multipoles for the same configurations. The pseudo multipoles agree with the PSP multipoles typically by 3 decimal places for the lighter atoms and 2 decimal places for the heavier atoms with filled d-shells.

• Energy differences between all pairs of these atomic configurations. Since accurate energy differences are our main goal, the energy differences between various electronic configurations of the atom have to agree to 1 kcal = 1.6 mHa or better.

We start by constructing non-relativistic PSPs, as it allows us to focus on minimizing pseudization errors without worrying about inconsistencies among different scalar-relativistic Hamiltonians used in practice and their implementations. Once the precision of these PSPs is verified, we use them as the initial guess for fitting the parameters in the scalar-relativistic case. For this purpose, we employ the AZORA Hamiltonian. We explain and justify this approximation in detail in Section 5.

The non-relativistic and AZORA PSPs are validated by comparison with the molecular all-electron calculations. This validation allowed us to discard some PSP's with insufficient accuracy while the all-electron data were not used for fitting the PSP parameters. In general, atomic orbitals with KS eigenvalues higher than −10 Ha have to be included in the valence.

We use the above considerations to generate PSPs for H to Br skipping the noble-gas elements. To ensure high precision, we use the number of valence electrons that is fully consistent with the LAPW settings used in this work. This number is larger than in typical production PSPs, and we did not investigate what is the necessary minimum of electron shells needed to achieve our precision target. The specific details on the core-valence partitioning is given in Table 1.

**Table 1:** Core-valence partitioning used for the MPC PSPs generated in this study

Element	Core	*Z*_ion_
H–F	—	1–9
Na–Cl	[He]	9–15
K–Br	[Ne]	9–25

## 2 Dipole moments

Employing the MPC approach leads to improved electron densities compared to ordinary norm conservation. To verify it, we calculate dipole moments of 18 selected small molecules in the isolated limit, as this quantity gives an insight into the quality of the density.^31^ The complete list of these molecules is given in Table 2, and the Computational Chemistry Comparison and Benchmarks Database hosted by the National Institute of Standards and Technology (NIST) provides their geometries that were obtained by geometry relaxations using a variety of exchange–correlation functionals and Gaussian-type orbitals (GTOs).^32^ For the present purposes, we select the PBE structure obtained with nominally the best basis set used for a given molecule and keep this structure fixed.

**Table 2:** Dipole moments (in atomic units) of isolated molecules calculated with all-electron (LAPW and GTO – Gaussian-type orbitals) and PSP approaches. The GTO and first LAPW entries correspond to non-relativistic data. The mean and maximum absolute percentage errors (MAPE and MaxAPE) are given with respect to the LAPW results

	LAPW^a^	GTO^a^	LAPW	MPC	GBRV	ONCV	HGH	kjpaw	rrkjus	JTH
CO	0.079	0.079	0.079	0.079	0.088	0.082	0.075	0.073	0.071	0.076
HF	0.695	0.694	0.693	0.694	0.702	0.701	0.693	0.701	0.702	0.703
HCN	1.162	1.163	1.161	1.160	1.162	1.175	1.159	1.169	1.169	1.163
H_2_O	0.710	0.709	0.708	0.708	0.708	0.716	0.710	0.714	0.716	0.714
NF_3_	0.089	0.089	0.089	0.090	0.092	0.087	0.083	0.093	0.093	0.096
NOF_3_	0.143	0.127^b^	0.143	0.145	0.159	0.152	0.134	0.146	0.146	0.149
COF_2_	0.393	0.393	0.393	0.392	0.371	0.394	0.394	0.388	0.387	0.384
NH_3_	0.585	0.584	0.584	0.583	0.588	0.597	0.587	0.589	0.590	0.585
NO_2_	0.105	0.105	0.103	0.104	0.103	0.104	0.103	0.106	0.106	0.106
O_3_	0.244	0.245	0.244	0.244	0.244	0.241	0.239	0.245	0.244	0.240
BeH	0.106	0.102	0.106	0.104	0.106	0.104	0.103	0.104	0.104	0.107
BeO	2.365	2.363	2.362	2.362	2.363	2.367	2.339	2.364	2.364	2.336
MgH	0.497	0.489	0.497	0.495	0.495	0.494	0.485	0.495	0.495	0.504
MgO	2.800	2.543^b^	2.794	2.791	2.795	2.797	2.723	2.789	2.789	2.763
ZnH	0.145	0.101^b^	0.132	0.131	0.131	0.128	0.134	0.131	0.132	0.132
ZnO	2.071	1.773^b^	2.010	2.010	2.005	2.018	1.985	2.001	2.003	2.005
CaH	0.960	0.961^b^	0.956	0.958	0.958	0.959	0.930	0.958	0.958	0.957
CaO	3.127	3.049^b^	3.109	3.104	3.108	3.113	3.016	3.107	3.108	3.106
MAPE (%)	—	0.5	—	0.4	2.0	1.6	2.2	1.4	1.5	1.6
MaxAPE (%)	—	3.5	—	1.5	12.2	5.9	7.5	7.5	9.7	7.0

a Non-relativistic calculations.

b Calculations with GTO basis sets of worse quality than aug-cc-pVTZ.

To generate reference values for the dipole moments as well as for further benchmarks, we use the all-electron LAPW+LO method as implemented in the electronic-structure code exciting.^33^ Interaction of periodic images is avoided by using the unit cells as large as 30*a*_0_ × 30*a*_0_ × 30*a*_0_ and introducing the spherical Coulomb cutoff in the Poisson solver. Table 2 shows that the dipole moments obtained using this approach are consistent with the NIST GTO data^32^ with the average and maximum absolute percentage errors being 0.5% and 3.5%, respectively. This comparison is made for non-relativistic results, and it includes only the molecules where the GTO calculations were performed using the highest-quality basis sets: either aug-cc-pVTZ, aug-cc-pVQZ or daug-cc-pVTZ. Among these molecules, the largest deviations of 3.5% and 1.5% correspond to BeH and MgH, respectively. If we exclude these outliers from the analysis, the average and maximum errors reduce to 0.1% and 0.5%, respectively. Such a level of agreement makes us confident about sufficient quality of the LAPW data.

For an assessment of the MPC PSPs, we provide scalar-relativistic all-electron data obtained using the atomic zero-order regular approximation (AZORA)^34^ and perform PSP calculations for the same set of isolated molecules using the QE code.^22^ As it does not support the spherical Coulomb cutoff in ground-state calculations, we extrapolate the dipole moments obtained at the cell sizes of *a* = 30*a*_0_ − 45*a*_0_ to the *a* → +∞ limit assuming the dipole moment converges to the isolated limit as O(*a*^−3^).

Table 2 shows the dipole moments from the MPC and other PSP calculations. We find that the MPC results show the best agreement with LAPW, as the average and maximum deviations are 0.4% and 1.5%, respectively. Furthermore, a mismatch above 1% is obtained only for 3 molecules: BeH, NF_3_ and NOF_3_. In contrast, the average and maximum deviations for other considered PSPs are 1.4–2.2% and 5.9–12.2%, *i.e.*, much larger than in the MPC case. The high accuracy of the MPC PSPs comes from the fact that the all-electron and PSP wave-functions coincide up to fairly small radii. The wavefunctions vary fairly rapidly near the nucleus, and, as a consequence, a very high energy cutoff is required when they are used within plane-wave codes.

## 3 Molecule set

The benchmark set for atomization energies consists of 669 electrically neutral molecules with s-, p- and d-elements from H to Po omitting noble gas atoms. The molecules are selected such that any given element is combined only with atoms from the first two rows of the periodic table of elements (PTE) covering a wide range of oxidation states. For example, the Cl atom is represented in 14 molecules with the oxidation state ranging from Cl^−1^ in HCl to Cl^+7^ in HClO_4_. The coordination numbers in molecules take on small values such as one or two and vary thus much more than in bulk systems. This, together with the strong variation of the oxidation states, ensures that our test set contains a very large variety of chemical environments. Only highly transferable PSPs should be able to describe all these environments accurately. The complete list of the molecules with their structures is provided in the dataset.^35^

We simulate these molecules in relatively small unit cells with the dimensions ranging from 5 × 5 × 7 Å to 7 × 7 × 7 Å chosen such that the periodic images of molecules have a separation of at least 3 Å. In this way, interactions between a molecule and its periodic images are weak, and the pseudization errors impact the atomization energy in the isolated (physical) and periodic (artificial) cases essentially to the same degree as we show in Section 4. The structural parameters, namely bond lengths, angles and dihedral angles, are fixed to the values given in ref. 32 and 36 for those molecules where the experimental structure are known. Typically, they contain the s- and p-elements, while data with structural parameters are scarce for molecules with d-elements. To satisfy the need for exploring the chemical space for such elements, we consider the molecules with the d-elements in hypothetical structures.

We refrain from considering isolated molecules as a benchmark for the following two reasons. The first is the intriguing fact that molecular atomization energies can not be calculated with guaranteed errors of less than 1 kcal mol^−1^ with the standard GTOs.^37^ Recently developed multi-wavelet codes^38,39^ can achieve this accuracy, but, at present, they are still lacking other features that are needed in this context such as various relativistic treatments. LAPW type codes can also reach this accuracy but need very large unit cells to approximate the free boundary condition characteristic for molecules making such reference expensive. The second reason is that also plane-wave codes become very slow when applied to molecular systems because also in this context very large periodic boxes are needed. We do not use real periodic solids as our test set either due to the above mentioned reduced variability of chemical environments as well as the frequently slow convergence of the calculations with respect to the *k*-point integration grids which can make bulk calculations quite slow.

By putting the molecules into fairly small periodic boxes, we avoid all these problems. Our artificial molecular structures exhibit the entire variety of chemical environments but both, all-electron as well as PSP calculations, can be done very rapidly. Since our benchmarks are synthetic, all the calculations are done only at the Γ-point. While full numerical convergence in a periodic problem formally requires a more extensive Brilloiun-zone sampling, using just 1 *k*-point consistently among all three employed periodic codes leads to fully comparable calculations in (semi)local DFT that allow us to assess the quality of the PSPs.

The dataset contains both closed- and open-shell molecules. In the latter case, the lowest-energy solution may be with broken symmetry despite a symmetric structure. For all used codes, we find that the self-consistency procedure does not necessarily converge to the same solution with different initial guesses yielding at least two different total energies. This effect introduces an undesired ambiguity, and it appears either due to the small size of the unit cells or small gap between occupied and unoccupied orbitals. To avoid it, we perform an explicit symmetrization of the density and the potential or employ predefined fractional occupation numbers for the for KS orbitals. The symmetrization is available in exciting and VASP, and this is the most straightforward approach for ensuring consistency. Such a functionality is not available in QE, and we therefore enforce occupation numbers. This workaround leads to solutions with symmetric densities in most cases, and it can be verified by inspecting the KS eigenvalue spectrum. Although our approach reduces the need for precise multipoles, it does not eliminate it entirely. For example, such a symmetrization of the OH molecule placed in a simulation cell along the *z* axis makes only the *x* and *y* directions equivalent. The *z* direction, however, is different from the other two in the context of the electron density assigned to the oxygen atom.

In the atomic calculations, we consider all elements in a simple cubic unit cell with the lattice constant of 5 Å and disallow spin polarization. As with the molecules, we ensure the uniqueness of the solution either by enforcing the spatial symmetry of the KS potential/density or employing preselected fractional occupations. Such a choice allows us to avoid solutions with broken symmetry that typically present a challenge to PSPs and introduce a constant error in atomization energies complicating further analysis. To illustrate this issue, we report the energy differences between the symmetric spin-restricted solution and the asymmetric spin-polarized lowest-energy solution. Table 3 shows these results for six chemical elements with a partially filled p shell. The MPC PSPs yield results close to the all-electron reference. The largest deviations of 0.24 kcal mol^−1^ and 0.27 kcal mol^−1^ are well within the chemical-accuracy threshold and are obtained for the O and F atoms, respectively. For the other considered PSPs, the largest errors either go beyond this threshold (ONCV and GBRV) or approach it (SSSP-precision). As the errors in the atomic references have no effect on energy differences between molecules, we leave this issue aside and calculate the atoms enforcing symmetry and ignoring the spin polarization.

**Table 3:** Energy difference (given in kcal mol^−1^) between the symmetric non-magnetic and lowest-energy solutions in the isolated limit. The data were obtained in all-electron (AE) and PSP (MPC, ONCV, GBRV and SSSP-precision) calculations

	AE	MPC	ONCV	GBRV	SSSP
B	−10.46	−10.47	−11.76	−11.26	−11.07
C	−31.71	−31.71	−33.74	−34.02	−31.73
O	−43.82	−43.58	−46.74	−46.80	−43.82
F	−15.96	−15.69	−15.07	−17.16	−15.09
S	−23.82	−23.75	−24.69	−24.21	−24.21
Cl	−7.56	−7.49	−7.81	−7.88	−7.60

The VASP code does not support user-defined occupation numbers making it impossible to remain consistent between all codes for all d^1^–d^8^ atoms except for Pd. In the VASP case, we perform spin-polarized calculations using a face-centered cubic cell. Our custom version of the exciting supports both options, and we provide reference in either case.

## 4 All-electron reference

The LAPW+LO method partitions space into atomic spheres and the interstitial region where wave functions are described by means of atomic-like orbitals and plane waves, respectively. Once the LAPW+LO basis is saturated in both regions, the total energy converges to the complete-basis limit.

We set the radii of atomic spheres to *r*_MT_ = 0.7, 1.0, 1.1 and 1.2 Bohr for the elements of the first, second, third and fourth to sixth periods, respectively. Such a choice avoids overlaps between the spheres in all molecules, and the maximum wavevector for LAPWs is fixed to *K*_max_ = 10 Bohr^−1^ in all calculations. It corresponds to the dimensionless LAPW cutoffs of *r*_MT_*K*_max_ = 7, 10, 11 and 12, respectively. This choice ensures a consistent level of precision for different elements, and cutoff ratios roughly match the suggestion given in ref. 40.

Previous studies showed that LAPW+LO as such allows us to reach agreement with the multi-resolution analysis better than 0.01 kcal mol^−1^ in terms of the absolute total energy in non-relativistic^41^ and ZORA calculations.^42^ In the present study, the precision requirements are more modest, hence we use lower LAPW cutoffs compared to those calculations. We nevertheless use the extensive LO sets (including high-energy LOs) for H–Cl prepared in ref. 41. For K and beyond, the prepared sets of LOs match those used in ref. 8. Our numerical tests show that these settings are sufficient to yield atomization energies that agree with the complete-basis limit well within 0.10 kcal mol^−1^.

To verify our LAPW+LO precision estimates, we perform an indirect comparison with the multiwavelets code MRChem.^39^ This DFT code employs a methodology with a systematic way of reaching the complete-basis limit by adaptive resolution. In the first calculation, we obtain errors in the atomization energies due to the MPC PSPs in a small box as described in Section 3. The LAPW+LO calculations serve as a reference, whereas the PSP calculations are performed using BigDFT^43^ and QE,^22^ based on the wavelet and plane-wave formalisms, respectively. We find that, in the converged limit, the two PSP codes, BigDFT and QE, yield atomization energies well within 0.10 kcal mol^−1^. In the second calculation, we obtain the errors for the same molecules using the free boundary conditions. This time, the reference and PSP data are acquired using MRChem and BigDFT, respectively. We employ consistent molecular geometries in both calculations. The different boundary conditions are selected, because periodicity is not supported in multiwavelets, whereas obtaining the total energies of molecules in the isolated limits means additional computational effort for LAPW+LO. Finally, this comparison is performed using the non-relativistic approximation, because relativistic Hamiltonians became available in the multiwavelet methodology only recently.^42^ Furthermore, it would restrict us to ZORA which is not the optimal choice for this type of a study as discussed below.

**Fig. 1:**
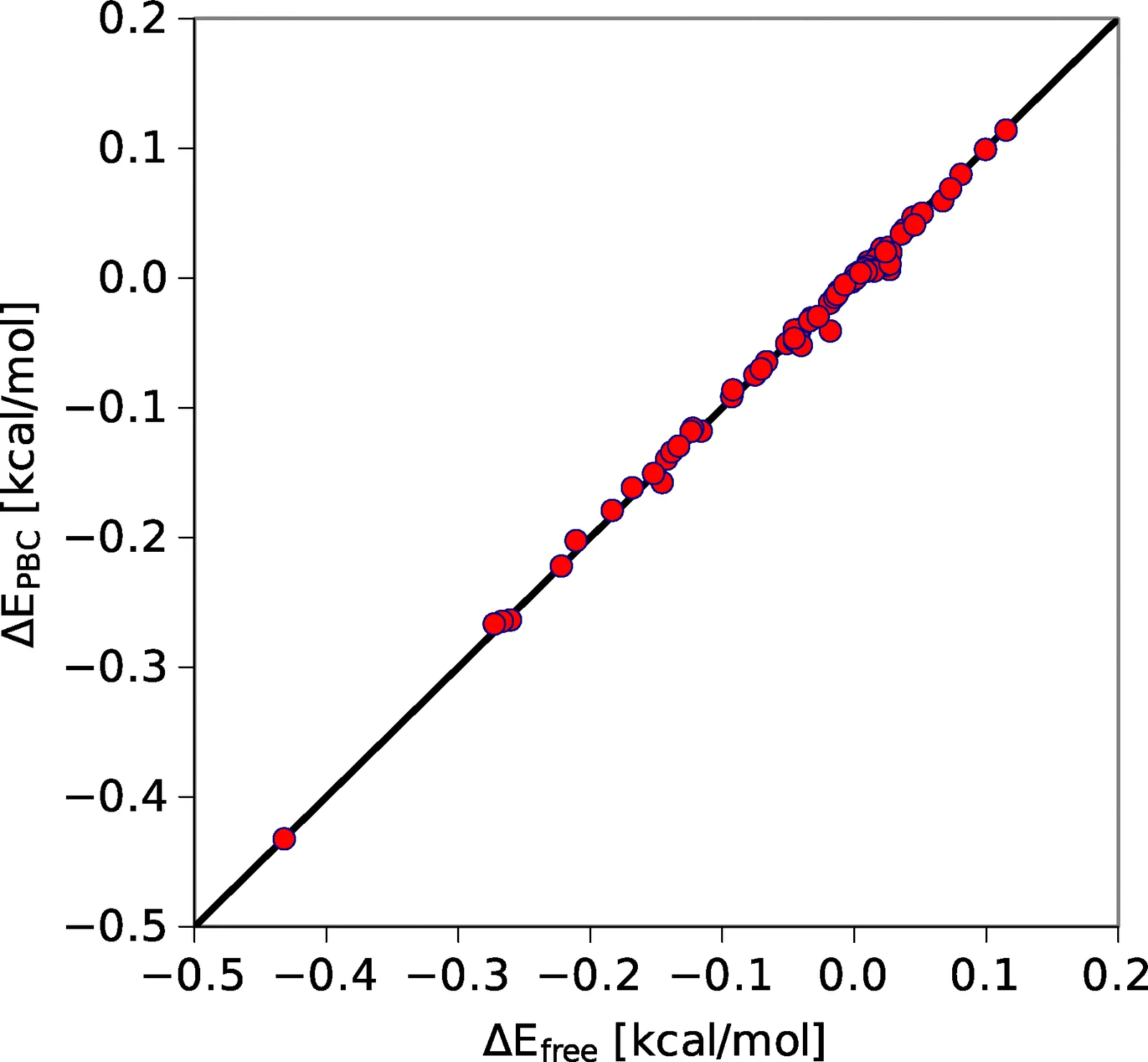
Errors due to the MPC PSPs in the atomization energy of 76 molecules (containing only the elements in the range H–F) in calculations with periodic and free boundary conditions labeled as “PBC” and “free”, respectively. The black line corresponds to the perfect agreement, namely, Δ*E*_PBC_ = Δ*E*_free_.

The errors obtained in these two calculations are presented in Fig. 1. The atomization energies obtained using the non-relativistic MPC PSPs for H to F agree with all-electron result within 0.50 kcal mol^−1^. The largest absolute error of 0.43 kcal mol^−1^ is obtained for N_2_O_3_. The errors in atomization energies for the molecules calculated with free and periodic boundary conditions are virtually identical with a largest deviation of 0.02 kcal mol^−1^. Based on these results, we conclude that (i) the chosen unit cells are sufficiently large for providing a high-quality estimate for the PSP errors, (ii) using the Γ-only calculations are fully sufficient, and (iii) our LAPW+LO calculations are appropriately converged.

## 5 Relativistic approximations

We provide all-electron reference and the MPC PSPs within the non-relativistic and scalar-relativistic approximations. Practical applications, however, require that at least scalar-relativistic effects are accounted for in calculations with most chemical elements. Moreover, a reliable description of molecules with sixth-row elements (especially 6p) requires considering the spin–orbit coupling. LAPW provides a reliable framework for including it and has been used as a reference tool in this context.^44^ Also, in the approach for constructing MPC PSPs, including spin–orbit coupling is possible.^18^ Nevertheless, we limit our scope to the scalar-relativistic case as it is a simpler problem where the PSP precision has not been fully solved yet.

We consider the following two approximations: the zero-order relativistic approximation (ZORA),^45,46^ atomic ZORA (or simply AZORA)^34^ and the infinite-order relativistic approximation (IORA).^47^

The kinetic energy operator within ZORA is expressed as3
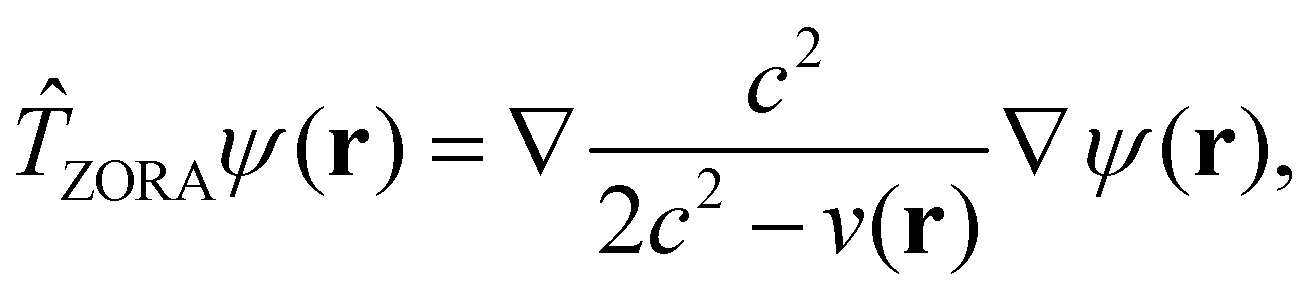
where *v*(**r**) is the effective KS potential. The total energy within this approximation varies if *v*(**r**) is shifted by a constant limiting ZORA's reliability. The source of discrepancies is the arbitrary additive constant in the electrostatic potential entering *v*(**r**). For finite systems, the electrostatic potential is defined in a unique and consistent way by setting it to 0 at the infinite distance from a molecule. Such an approach is impossible for periodic systems, and, in practice, the additive constant is determined by technical details. In an LAPW calculations, they include the cell volume and pseudocharges in atomic spheres^48^ making it difficult to ensure that different total energy calculations are consistent for evaluating atomization energies.

There are several approaches for resolving the issue described above, including scaled ZORA, first-order regular approximation^46^ and AZORA. The latter one is a simple yet established workaround,^8,49^ and we employ it without exploring the other methods. AZORA replaces *v*(**r**) with a predefined model potential *v*_at_(**r**), and the kinetic energy reads4
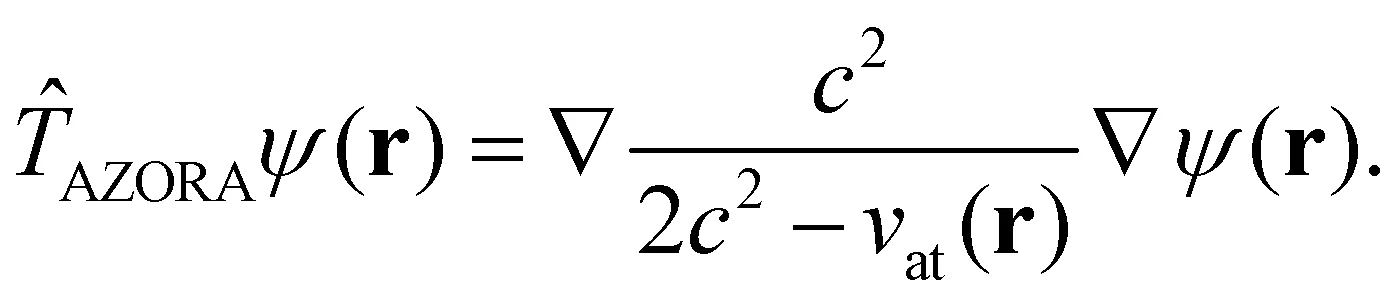
*v*_at_(**r**) is typically chosen as a self-consistent KS potential obtained for an isolated neutral atom, but the exact details of how it is done depends on a DFT code used. In the atomic solver used for constructing MPC PSPs, we apply the atomic potential in the entire space. In LAPW calculations, we apply eqn (4) solely within atomic spheres and disregard contributions from the interstitial region. Such an approach is justified because the main contributions to the relativistic corrections arise from the regions close to nuclei. Furthermore, neglecting the interstitial contributions allows us to avoid calculating a superposition of atomic potentials.

As another solution for improving upon ZORA, we use IORA which incorporates an additional term in the kinetic energy in the following manner:5
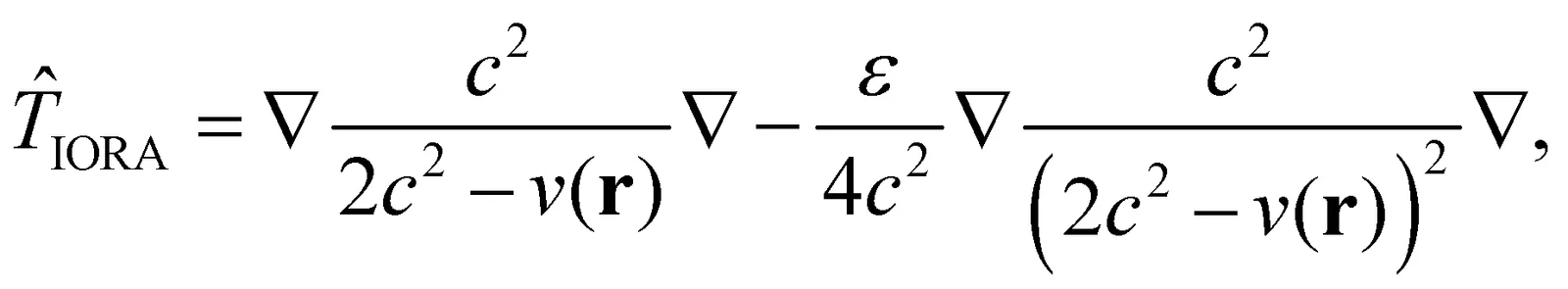
where *ε* is the KS eigen-energy. IORA solves the issues with the dependence on the potential shift nearly completely, as the potential shift has no noticeable impact on atomization energies. Noteworthy, the solutions of the scalar-relativistic Schrödinger equation with *T̂*_IORA_ are not orthogonal due to the term linear in *ε*. Orthogonality is restored if the small components are considered. However, their contribution to the total energy is negligible in the case of valence and semicore electrons. For this reason, we consider only the large components in this case. The core electrons in LAPW calculations are treated differently due to their confinement in atomic spheres where the four-component Dirac equation is solved in this case.

**Fig. 2:**
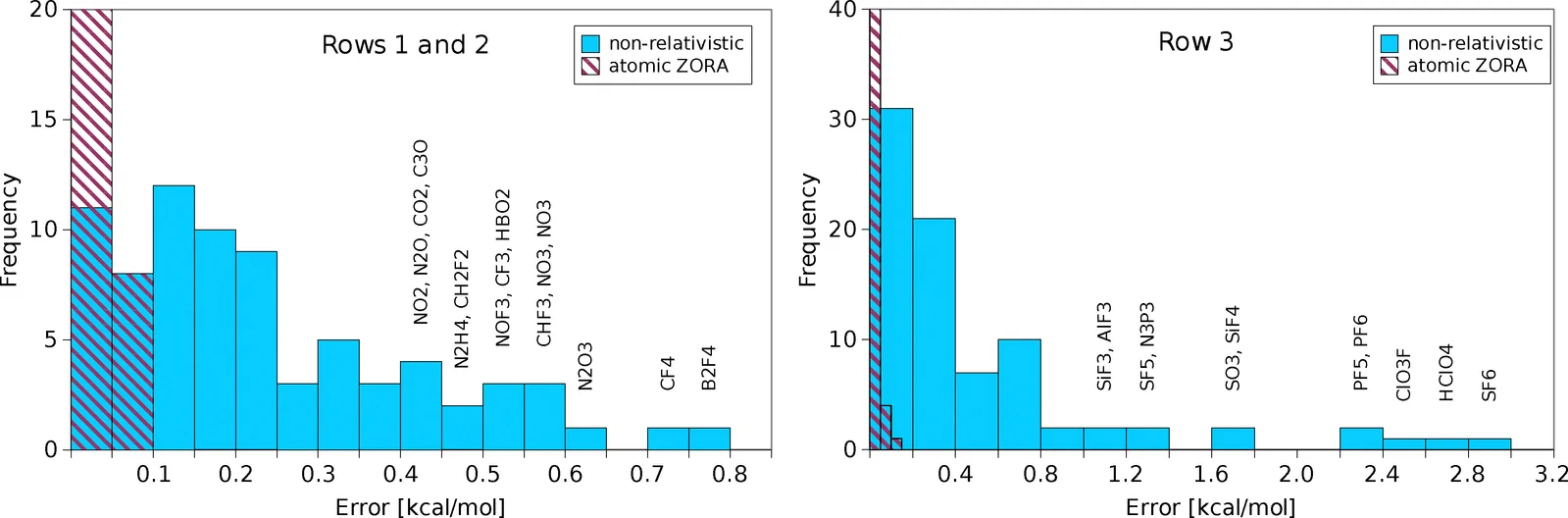
Comparison of errors in atomization energies of molecules obtained using the non-relativistic approximation and AZORA. The reference values are obtained with IORA.

To understand which relativistic approximations are sufficiently accurate for benchmarking purposes we perform atomization-energy calculations. First, we consider molecules from the first three rows of the PTE. The results summarized in Fig. 2 show that atomization energies obtained with AZORA and IORA are within 0.10 kcal mol^−1^ except for HClO_4_ for which we obtained the difference of 0.11 kcal mol^−1^. Agreement between the two approximations makes us confident that either of them is suitable for providing an all-electron reference and generating PSPs.

Neglecting relativistic effects changes the atomization energies by up to 0.8 kcal mol^−1^ for molecules containing elements from the first two rows of the PTE only. For the molecules containing atoms from the third row, non-relativistic calculations cannot guarantee this level of accuracy for 11 molecules out of the considered 82. Note that the largest errors are obtained for molecules with elements in high oxidation states.

**Fig. 3:**
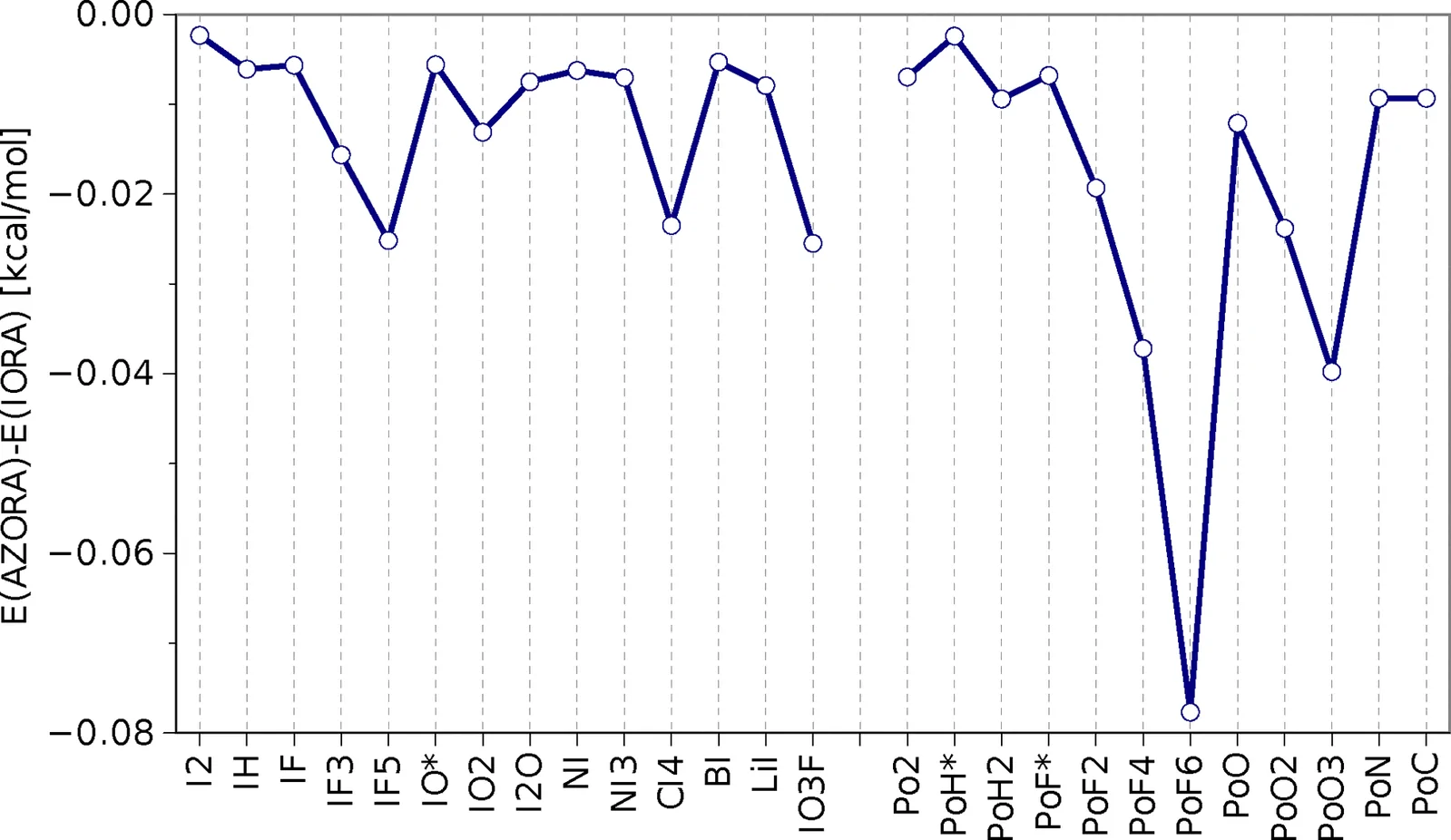
Difference of atomization energies obtained using the AZORA and IORA Hamiltonians.

The complete molecule set, however, contains heavy elements too, and it is important to verify that our relativistic model is sufficiently reliable in all cases. Fig. 3 compares the IORA and AZORA atomization energies obtained for molecules with I and Po, the heaviest elements of the rows 5 and 6, respectively. We find that both approximations yield the results within 0.1 kcal mol^−1^, and, yet again, the largest differences are observed for molecules with either I or Po in their highest oxidation states. The consistent results indicate that the chosen relativistic models are sufficiently accurate for the purposes of this work.

## 6 Atomization energies

### 6.1 Multipole-conserving pseudopotentials

We assess the numerical performance of the MPC PSPs using QE. Owing to the choices made for the core-valence partitioning and parametrization constraints, these calculations require large basis sets. Wave functions and the KS potential are represented with plane waves with the cutoff energy of *E*^wf^_cut_ = 1500 Ry and *E*^pot^_cut_ = 6000 Ry, respectively. These settings are excessive for most chemical elements. Cutoffs this high are mainly required for second-row elements, especially O and F. Since a large part of our dataset is oxides and fluorides, these settings are used for all molecules. We note that the cutoff requirements for MPC PSPs are significantly higher than those of typically used PSPs, and it impacts computational cost. Limiting the discussion to large systems and a plane-wave basis as used in QE, the most time is spent in the orthogonalization carried out in the eigensolver. The corresponding effort scales as O(*N*_el_^2^*N*_PW_), where *N*_el_ and *N*_PW_ ∼ (*E*^wf^_cut_)^3/2^ are the numbers of electrons and plane waves, respectively. In practical terms, containing the basis-incompleteness error to 0.1 kcal mol^−1^ for a Br atom using 25-electron MPC and 17-electron ONCV PSPs requires *E*^wf^_cut_ = 800 Ry and 80 Ry, respectively. With these settings, we expect roughly a 70-fold increase in the effort. If, on the other hand, these pseudopotentials are used in combination with an adaptive basis set such as multi-wavelets or numerical atomic orbitals, the number of basis functions increases only moderately. The main reason for an increase in computing time is in such a case then the larger number of electrons. Depending on the algorithm for the wave function optimization, the scaling with respect to the number of electrons can vary from linear up to cubic.^50^ Going from a 17 electron to a 25 electron PSP would then in the worst case result in a three-fold increase of the computing time.

**Fig. 4:**
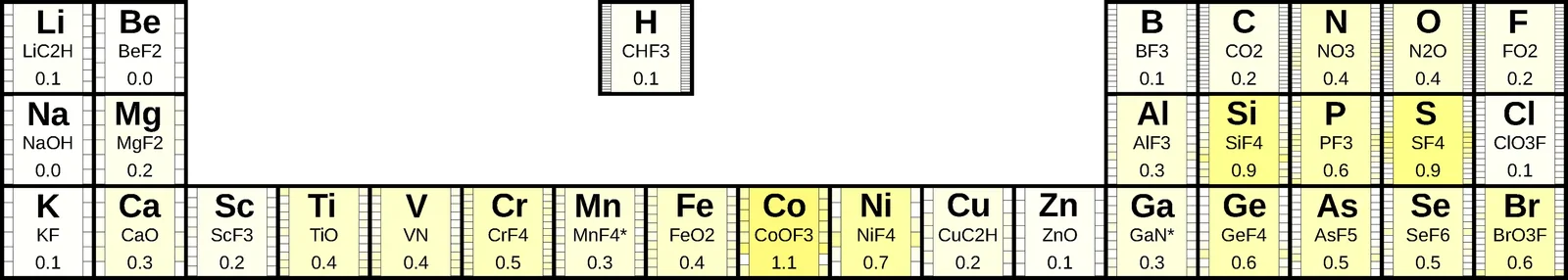
Errors in atomization energies due to the scalar-relativistic MPC PSPs. The color-coding and the layout are explained in Fig. 5. The color of the tiny cells on the right-hand side corresponds to the total error divided by the number atoms of a given kind.

**Fig. 5:**
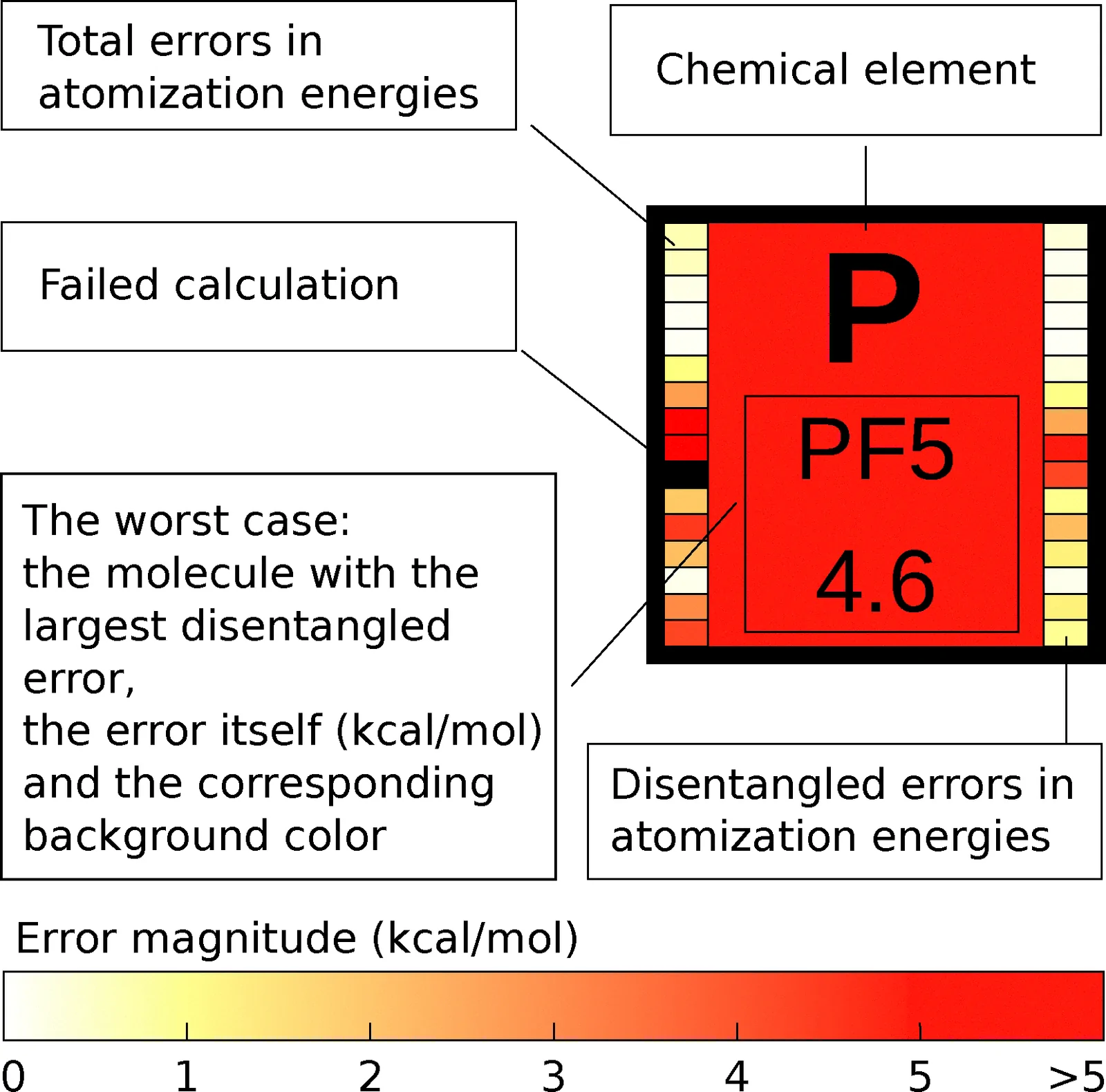
Notation used in the diagrams with the periodic table of elements. Molecules marked with a star correspond to a calculation with enforced fractional occupation numbers.

The errors in the MPC atomization energies with respect to the all-electron data are summarized in Fig. 4 in the shape of a PTE. The numerical data are given in ref. 51 and the raw data for all calculations are available in the dataset.^35^ The layout of the PTE is explained in Fig. 5. Among the molecules comprising elements from first 4 periods, the largest absolute deviation from all-electron atomization energies is 1.1 kcal mol^−1^ obtained for the hypothetical CoOF_3_ molecule where Co is in its highest observed oxidation state Co^5+^. In all other cases, the error is below 1 kcal mol^−1^ thus the MPC PSPs comply with chemical accuracy. The diagram in Fig. 4 shows the molecule for which the largest error is obtained for a given element. In many cases, we find that the most challenging molecule corresponds to the highest considered oxidation state as it is in the Co case.

Due to the extensive use of the elements from the first two rows in the entire test set, we pay a particular attention to the atomization energies of 78 molecules comprising exclusively these elements. In this case, the largest absolute total error of 0.55 kcal mol^−1^ is obtained for N_2_O_3_ as in the non-relativistic case. The errors under 0.10 kcal mol^−1^ and 0.20 kcal mol^−1^ appear in 75% and 90% of cases, respectively.

The molecules from the subset contain 2–6 atoms, and it is natural to expect worse atomization energies for larger molecules. For this reason, we also provide the errors per atom of a given kind assuming all-electron quality for the other kinds. For example, if the analyzed PSP in the N_2_O_3_ calculation is for the N(O) atom, this normalized error is 0.28 (0.18) kcal mol^−1^, *i.e.*, one half (third) of the total one. This is clearly not a decomposition into atomic contributions, but rather their conservative estimate. Fig. 4 shows also these quantities and highlights the worst case for each molecule. Although the largest normalized error is 0.44 kcal mol^−1^ (in the NO_3_ case considering the PSP for the N atom), this quantity for a given element from the first two rows is under 0.10 kcal mol^−1^ and 0.20 kcal mol^−1^ in 84% and 95% of cases, respectively. Therefore, we conclude that the MPC PSPs for these elements yield essentially all-electron quality for atomization energies.

### 6.2 Open-source production pseudopotentials

We next analyze commonly used open-access PSPs with a quality score of *Δ* ≤ 1.0 meV atom^−1^ in ref. 8. A strict definition of the *Δ*-value is given in ref. 14, but, loosely speaking, this quality measure is the root-mean-square error between two equations of state obtained for elemental solids using a tested PSP and the LAPW reference. Ref. 8 estimates the *Δ* value of 1 meV atom^−1^ as the level of uncertainty in equation-of-state data comparing different experimental studies.

**Fig. 6:**
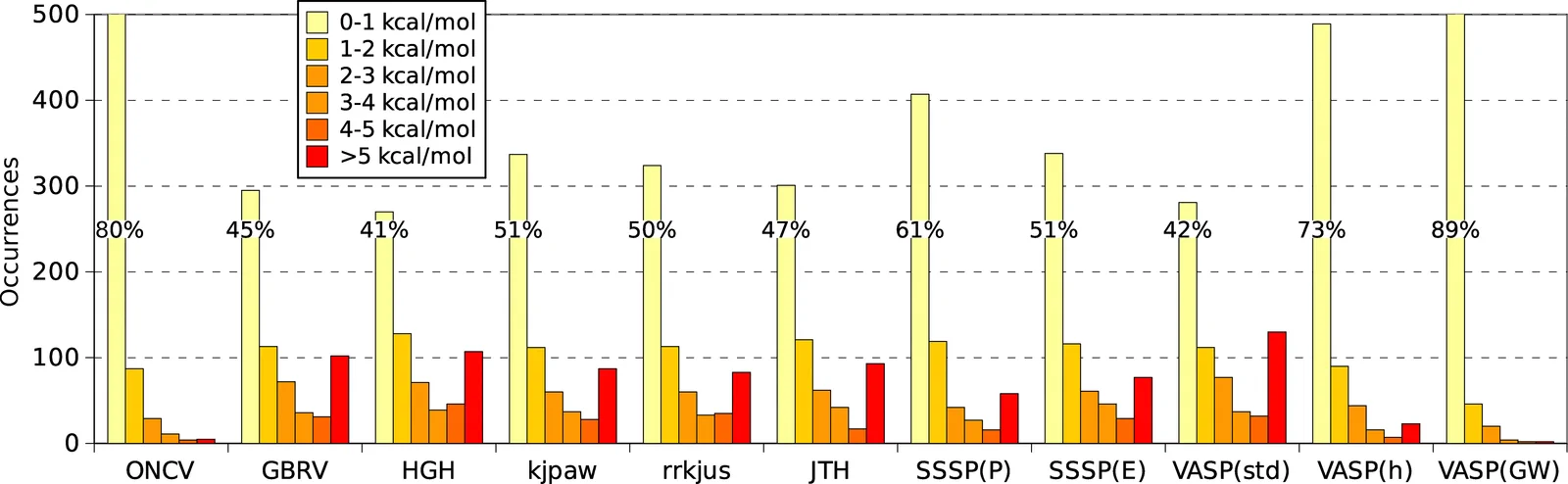
Distribution of errors in total atomization energies (kcal mol^−1^) for the considered PSPs. The given percentages correspond to the fraction of molecules for which chemical accuracy (error below 1 kcal mol^−1^) with respect to all-electron data was attained.

**Fig. 7:**
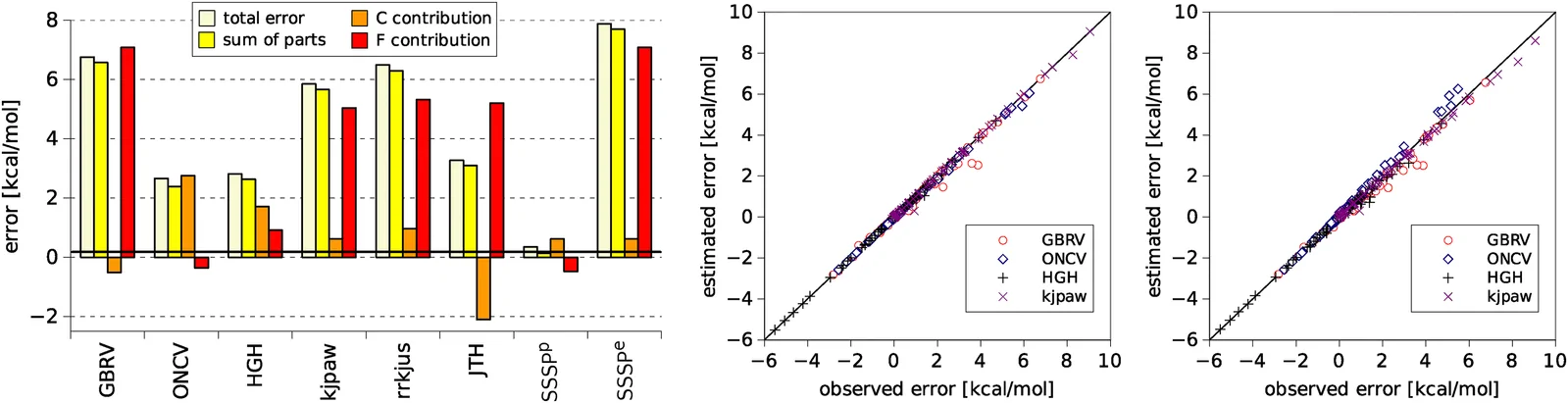
On the left-hand side, errors in atomization energies for the CF_4_ molecule using 8 families of PSPs. The total errors are given alongside with estimates of the contributions from the C and F atoms according to eqn (8) and their sum. The black line corresponds to the total error in the calculation with multipole-conserving PSPs. In the middle, calculated *versus* reconstructed errors in atomization energies according to eqn (7). 68 molecules with elements from the first two periods are considered. On the right hand-side, calculated *versus* reconstructed errors in atomization energies as in eqn (8).

As in the MPC PSP case, we carry out calculations using the QE code and using the same computational settings. The raw data (inputs and outputs) are available in ref. 35, and detailed results are given in the SI.^51^Fig. 6 shows the distribution of deviations from the all-electron reference obtained for considered PSPs, and the percentage of errors above the chemical-accuracy threshold ranges from 20% to 59%. Nearly all such molecules are heteronuclear which makes the result difficult to interpret, as it is not immediately clear how to attribute errors to specific atoms. As an illustration, we consider the CF_4_ molecule. The total error in the atomization energy is in the range of 0.35–7.88 kcal mol^−1^ depending on the PSPs (see the left panel of Fig. 7). Which chemical species (C or F) needs to be described better to improve the PSP performance?

Considering a heteronuclear diatomic molecule AB, we introduce the following Ansatz for the error in the atomization energy:

6 Δ*E*(A^PSP^B^PSP^) = Δ*E*(A^PSP^B^AE^) + Δ*E*(A^AE^B^PSP^),

where the labels “PSP” and “AE” show whether the atoms A and B are described using a given PSP or an all-electron method. Eqn (6) assumes that the errors due to applying the PSPs for the atoms A and B are independent and can be assigned to Δ*E*(A^PSP^B^AE^) and Δ*E*(A^AE^B^PSP^), respectively. It is a good approximation if the PSPs introduce just a small perturbation to the valence electronic structure.

Although the all-electron and PSP methods were already combined in a multi-wavelet code,^52^ that implementation supported only PSPs in the dual-space Gaussian form. This limitation makes the decomposition of errors based on eqn (6) currently unavailable for other PSP types, and, instead, we consider A^PSP^B^MPC^ and A^MPC^B^PSP^ instead of A^PSP^B^AE^ and A^AE^B^PSP^, respectively. Using eqn (6) to express Δ*E*(A^PSP^B^PSP^), Δ*E*(A^PSP^B^MPC^), Δ*E*(A^MPC^B^PSP^) and Δ*E*(A^MPC^B^MPC^), we arrive at the following expression:

7 Δ*E*(A^PSP^B^PSP^) = Δ*E*(A^PSP^B^MPC^) + Δ*E*(A^MPC^B^PSP^) − Δ*E*(A^MPC^B^MPC^),

It is an identity that follows directly from eqn (6), and it can be generalized to molecules beyond diatomic ones. The middle panel of Fig. 7 compares the left- and right-hand sides of eqn (7) labeled as observed and estimated errors, respectively. The agreement is excellent in a vast majority of cases, and it justifies the assumption that the errors due to individual atoms are additive as given in eqn (6). The major outliers correspond to atomization energies of HF, H_2_O_2_ and H_2_O obtained using GBRV. We argue that the outliers are a consequence of overlapping projectors in these ultrasoft PSPs used for the molecules with very short bonds: O–H and H–F.

According to our numerical experiments with the MPC PSPs, Δ*E*(A^MPC^B^MPC^) ≈ 0, and we simplify eqn (7) to

8 Δ*E*(A^PSP^B^PSP^) ≈ Δ*E*(A^PSP^B^MPC^) + Δ*E*(A^MPC^B^PSP^).

The two remaining terms on the right-hand side have a straightforward interpretation and allow us to estimate the errors due to individual atoms directly. The right panel of Fig. 7 compares the left- and the right-hand sides of eqn (8). The obtained deviations are larger than in the case of eqn (7), however, they are sufficiently small to conclude that eqn (8) provides reasonable estimates of the errors due to individual atoms.

Eqn (8) provides now also the basis for determining individual error contributions from PSPs of different elements in a molecule to the total atomization error. First, one performs a calculation where all the elements are described by their MPC PSPs. Then one replaces one elemental PSP after the other by the PSP to be tested. The difference in energy is then the PSP error for this element and the sum of all these energies is to a very good approximation equal to the atomization error.

As an illustration, we apply this analysis to the CF_4_ molecule. The total error in the atomization energy is given by Δ*E*(C^PSP^F_4_^PSP^) per definition. To decompose it into the atomic contributions, we calculate Δ*E*(C^PSP^F_4_^MPC^) and Δ*E*(C^MPC^F_4_^PSP^). The left panel of Fig. 7 displays these total and partial errors for 8 different PSP families. As a result of this analysis, we find that for ONCV and HGH the dominant source of the error is a PSP for the C atom, whereas, for GBRV, kjpaw, rrkjus and JTH, it is the F atom. This result shows the importance of being able to perform such an analysis, because it provides insights into which PSP requires an improvement or which PSPs should be combined to assemble optimal sets of PSPs taken from different families. SSSP(precision) and SSSP(efficiency) are such assembled sets, and they correspond to C^kjpaw^F_4_^ONCV^ and C^kjpaw^F_4_^GBRV^, respectively. The selected combination for SSSP(precision) clearly shows the optimal performance.

We notice that Δ*E*(C^JTH^F_4_^MPC^) and Δ*E*(C^MPC^F_4_^JTH^) have opposite signs, and the total error in the JTH calculation is above the chemical accuracy threshold, yet it is not excessively large. This cancellation of errors may mislead us if the decomposition is not performed. The total errors, thus, are less informative, and we focus on individual atomic contributions in the rest of this paper.

**Fig. 8:**
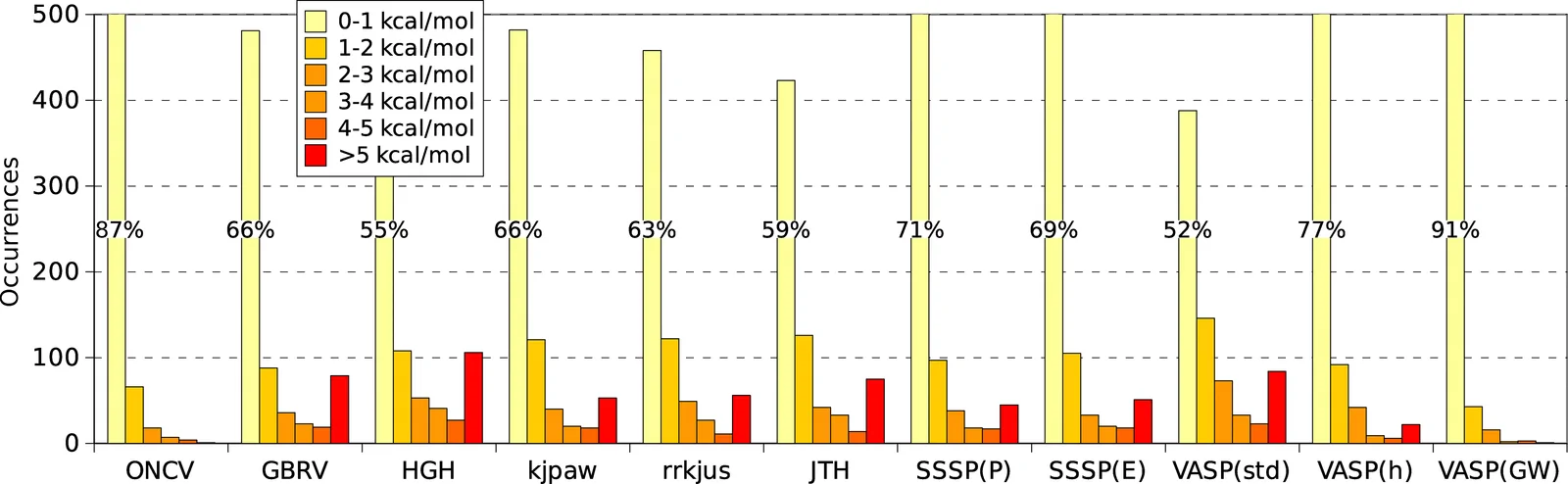
Distribution of errors in atomization energies (kcal mol^−1^) decomposed into individual atomic contributions. The given percentages correspond to the fraction of cases for which chemical accuracy (error below 1 kcal mol^−1^) with respect to all-electron data was attained.

Fig. 8 shows the distribution of the decomposed errors obtained for the considered PSPs. This diagram takes into account 747 atomization-energy errors calculated for 669 molecules. The additional 78 tests appear due to heteronuclear molecules containing exclusively light elements (H–F). Each of these molecules contributes to the overall statistics multiple times. We find that the percentage of the decomposed errors above 1 kcal mol^−1^ ranges from 13% to 45%. It is lower and more representative than in the case with total errors, as the light elements do not contribute to the statistics obtained for the heavy ones. Nevertheless, we find that none of the open-access PSP families guarantees atomization energies with absolute decomposed errors within 1 kcal mol^−1^.

**Fig. 9:**
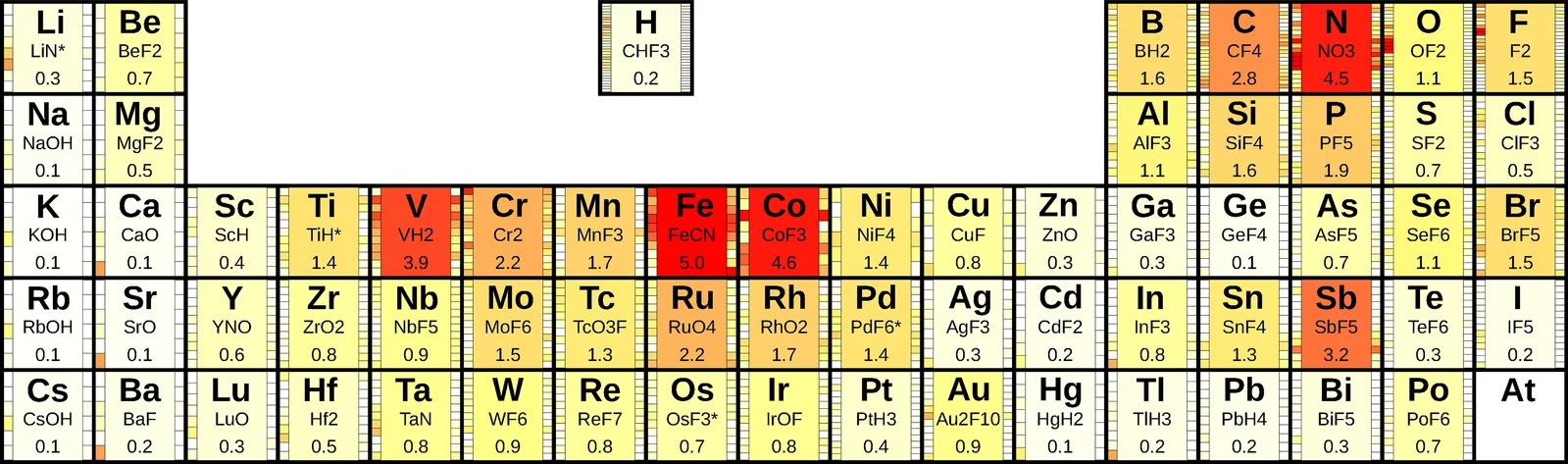
Errors in atomization energies due to the ONCV (version 0.5) PSPs. The color-coding and the layout are explained in Fig. 5.

**Fig. 10:**
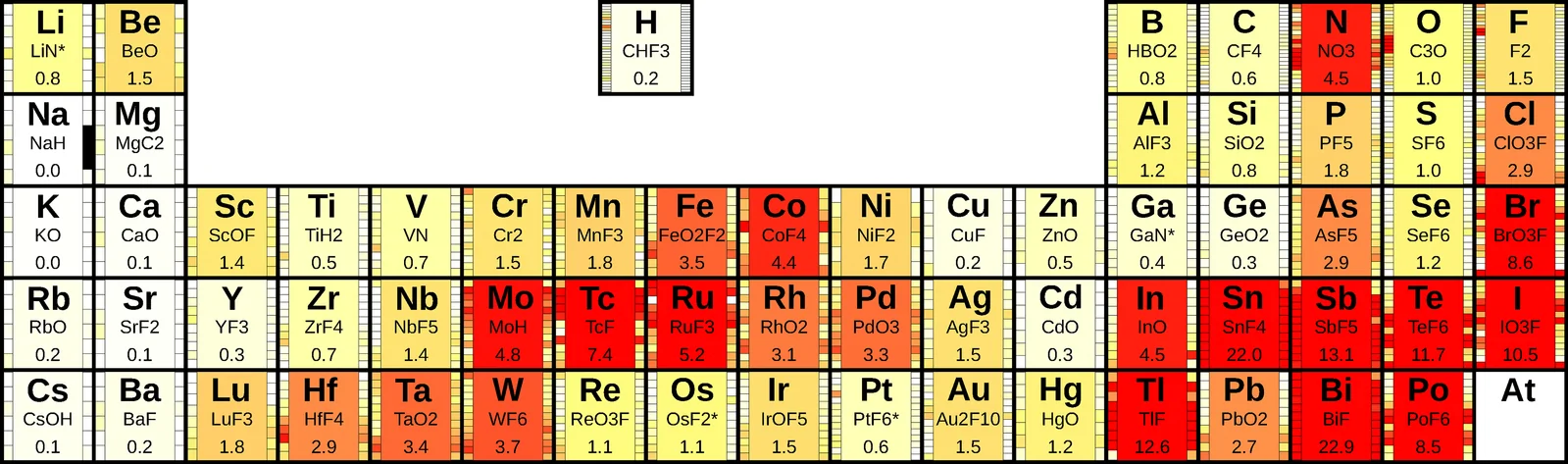
Errors in atomization energies due to the SSSP (precision, version 1.3.0) PSPs. The color-coding and the layout are explained in Fig. 5.

Fig. 9 and 10 provide a more detailed insight into the errors in atomization energies for the ONCV (version 0.5) and SSSP (precision, version 1.3) PSPs. Diagrams for further PSPs are available in the SI.^51^ If some molecule A_*x*_B_*y*_C_*z*_ is considered while focusing on the element A, the diagrams provide the total and decomposed errors Δ*E*(A^PSP^B^PSP^C^PSP^) and Δ*E*(A^PSP^B^MPC^C^MPC^)/*x*, respectively.

Typically, the most difficult cases corresponds to the highest oxidation states along a series of molecules (*e.g.* from HCl to HClO4. For all PSP families, except for ONCV, 4p-, 5p- and 6p-elements present a challenge. For example, the range of the largest disentangled errors obtained for Te (always in the TeF_6_ molecule) is 11.7–50.3 kcal mol^−1^ compared to 0.3 kcal mol^−1^ in the ONCV calculation. Such a difference can be explained by choices made for core-valence partition. Only ONCV PSPs includes 24 valence and semicore electrons corresponding to the atomic configuration 4s^2^4p^6^4d^10^5s^2^5p^4^, whereas all other PSPs are limited to 6 valence electrons (5s^2^5p^4^). Semi-core shells are most exposed in high oxidation states and therefore also more sensitive to the chemical environment. We also point out that energy differences, such as atomization energies, generally benefit from cancellation of errors between the individual total-energy calculations from which they are formed. If these individual calculations are done for molecules with atoms in very different oxidation states, for example, Te^0^ and Te^+6^, the cancellation of errors becomes less reliable.

The PSPs do not comply with chemical accuracy for most transition metals too, although they are less challenging than the p-elements discussed above. The largest errors are obtained typically for molecules with a transition element in a high oxidation state, usually fluorides or oxides. However, there are exceptions such as the Mn atom, for which the most challenging molecules are MnF_3_ (for ONCV and GBRV) and MnH (for HGH, kjpaw, rrkjus and JTH). These are open-shell molecules with a spin polarization, and our tests show that the errors depend on the magnetic moment. Partially filled d-shells result in a non-spherical potential in the core region, implying that high-quality PSP calculations require reducing the size of the core region. Increasing the number of valence electrons in PSPs for the p-elements and adding the f-projectors for the d-elements are further potential measures that make calculations more expensive, yet they are likely useful for improving the quality of calculations. The open source PSPs have typically no f-projectors for the 3d and 4d elements, but the more accurate VASP GW PAWs have them.

We emphasize that these benchmark results are obtained in PBE calculations using PSPs generated with the same exchange–correlation functional. Strictly speaking, employing a different exchange–correlation functional formally requires a different set of PSPs. They are readily available for some PSP families, for example, there are ONCV, kjpaw and rrkjus parametrizations carried out with the PW92^53^ and PBEsol^54^ functionals. However, it is impossible to prepare PSPs for all possible cases, and therefore it is common to use the same PBE PSPs, for example, in calculations with hybrid functionals. We anticipate that the cancellation of errors mentioned above should be beneficial also in this case, nevertheless the atomization energies should deteriorate further in the worst-case scenarios.

The molecule set provides not only means of numerical assessment, but also a stress test for PSPs. The vast majority of calculations reach a successful finish, but there are exceptions marked with black tiny cells or blank element cells in Fig. 9 and 10. The reasons for the failed calculations are (i) inability to converge to self-consistency in 100 iterations, (ii) a termination due to linear dependence (failed Cholesky decomposition) while solving an eigenproblem, (iii) unwanted asymmetric solution. The issue (ii) stems from a choice of projectors in PSPs, and we encountered this problem for JTH (Si, Se and Lu), kjpaw (Ba, W) and rrkjus (Ba, W). The issues (i) and (iii) are somewhat related, as QE does not support symmetrization of densities or potential.

**Fig. 11:**
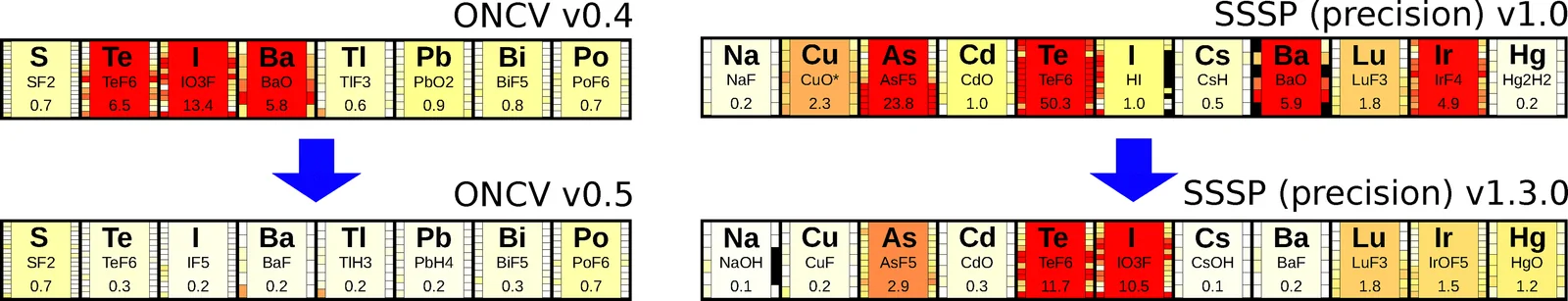
Impact of an update of PSPs (ONCV on the left, SSSP on the right). The color-coding and the layout are explained in Fig. 5.

Our analysis allows us to assess the improvements made as a result of the study in ref. 9. That work studied transferability of PSPs using a test set consisting of real and hypothetical oxides corresponding to a range of oxidation states. As a result of that study, Bosoni *et al.* suggested updates for the ONCV and SSSP families where PSPs for selected elements were adjusted. Fig. 11 summarizes the performance of the old and the new versions of the PSPs. In the case of ONCV, we find major improvements for Te, I and Ba. For the other, elements we observe either a slight improvement or no change in the numerical performance. Also for the SSSP (precision) PSPs, we find a major improvement for several elements: Cu, As, Cd, Te, Ba and Ir. The errors increased for Hg, and it appears so for I too. We note, however, that the old selection for this PSP combined with the MPC PSPs leads to crashing calculations for molecules with iodine in high oxidation states where the highest errors are expected. These issues disappear with the new PSP, as it performs robustly albeit with high errors for several molecules.

**Fig. 12:**
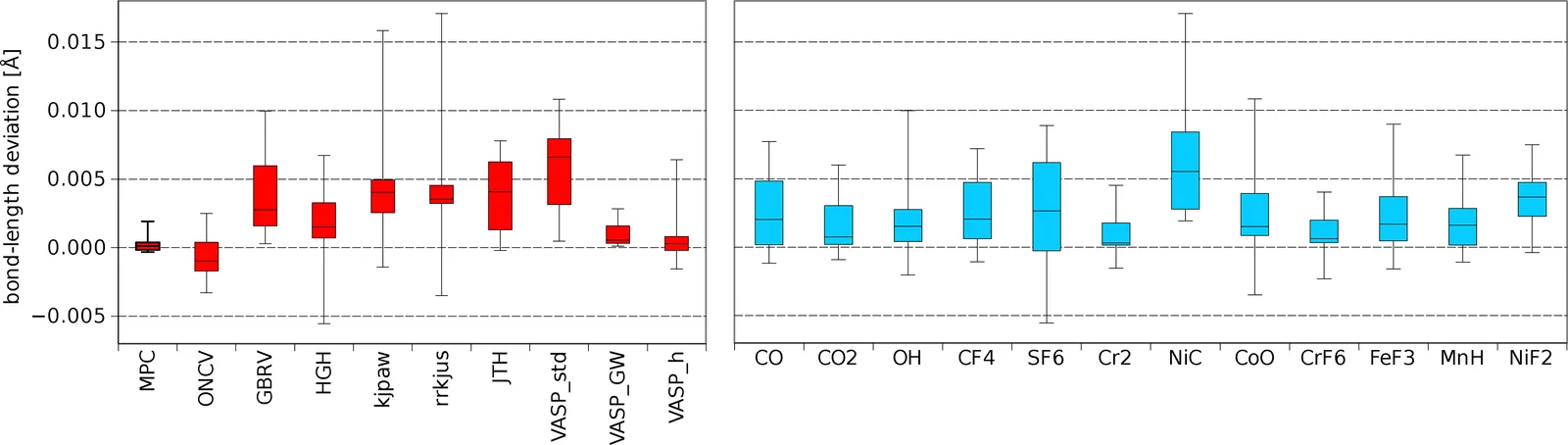
Distribution of PSP errors in bond lengths. The left and right panels show the distributions for each PSP and molecule considered, respectively. The box and whiskers show the minimum, first quartile, median, third quartile, and maximum of the distribution.

The discussion in this section is based on atomization energies, however, PSPs impact bond lengths and other properties. To compare the considered PSPs in this regard, we optimize the structure of twelve molecules (see the right panel of Fig. 12) with just one degree of freedom due to their symmetry. These molecules represent different potentially difficult cases: short bonds, high oxidation states and spin-polarization. Fig. 12 shows a distribution of errors in bond lengths obtained for each PSP with respect to all-electron results. We find that the MPC PSPs calculations reproduce the LAPW results remarkably well for all molecules with the largest deviation of 1.9 × 10^−3^ Å for NiC. For other PSPs, we find more significant discrepancies ranging up to 0.017 Å once again for NiC in a rrkjus calculation.

### 6.3 VASP PAW sets

We consider three groups of VASP PAWs sets: VASP_std – the same PSPs as used in the Materials Project,^4^ VASP_h – a selection of hard and small-core PSPs, VASP_GW – GW-ready PSPs (choosing the hard and small-core option whenever possible). The specific list of PAW sets is given in the SI.^51^ The analysis performed here slightly differs from that in the previous subsection. First, VASP implements PSPs and PAWs differently compared to QE and other plane-wave codes, and the MPC PSPs cannot be used here. For this reason, we disentangle the total errors using a compilation of the best performing VASP_h and VASP_GW PAWs for the elements of the first two periods. These PAWs yield the total errors within 1 kcal mol^−1^ for all molecules consisting of elements from the first two periods excluding BeF_2_. Second, VASP does not support predefined occupation numbers, however, it supports symmetry. This feature changes our approach for calculating the atomic reference for transition elements as explained in Section 3.

**Fig. 13:**
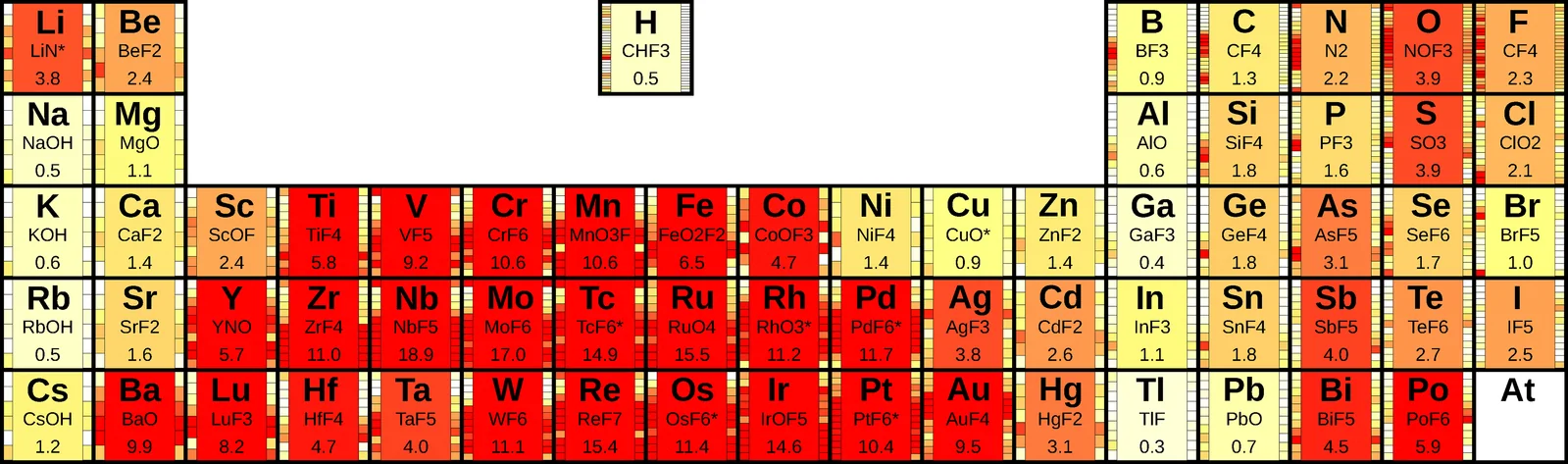
Errors in atomization energies in calculations with VASP PAW datasets as used in the Materials Project. The color-coding and the layout are explained in Fig. 5.

**Fig. 14:**
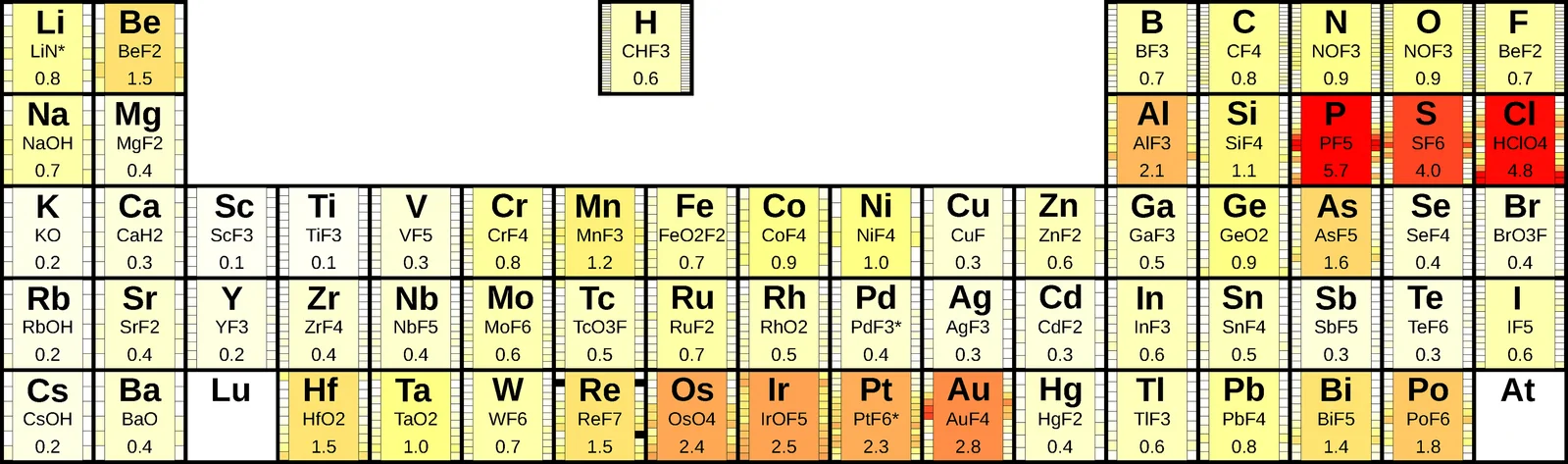
Errors in atomization energies in calculations with GW-ready VASP PAW datasets. The color-coding and the layout are explained in Fig. 5.

Fig. 6 and 8 summarize the overall performance of the considered VASP's PAW datasets. Again, we find broad ranges of the total and decomposed errors varying within 11–58% and 9–48%, respectively. More detailed is provided in Fig. 13 and 14 for VASP_std and VASP_GW, respectively. In the VASP_std calculations, the largest errors are obtained for the transition metals and are above the chemical accuracy threshold for the majority of the molecules with these elements. We find a much better numerical performance in general when the VASP_h and, especially, VASP_GW PSPs are used. Neither of these two sets guarantees the 1.0 kcal mol^−1^ precision for all elements. Picking the best PSP for each element allows one to compile a set, where this precision level is achieved for a large part of the PTE. In comparison to VASP_std, calculations using VASP_h and VASP_GW lead to somewhat higher computational expenses which is the price for precise calculations.

## 7 Conclusions

In conclusion, we have compiled a synthetic benchmark of 669 molecular structures with 65 different chemical elements. This test set aims at chemical diversity and covers a wide range of oxidation states for each element. Our database provides highly precise atomization energies for these molecular structures, and we have used it to assess the performance of widely used and our newly developed MPC pseudopotentials. The MPC pseudopotentials conserve not only the norm of the charge, but reproduce also higher multipole moments with much smaller errors than standard pseudopotentials. We have constructed such pseudopotentials for the elements of the first four periods. The obtained results are well within the chemical-accuracy threshold compared to our highly precise all-electron calculations. Our MPC pseudopotentials are quite hard and will therefore probably not be used widely in plane wave codes. They can however be used in the context of plane wave codes to establish the accuracy of other softer pseudopotentials for a system of interest. Our approach is actually able to decompose the error in total energies into individual atomic contributions and to detect in this way low quality pseudopotentials. This approach revealed that commonly used state-of-the-art pseudopotentials are still not fully compliant with chemical accuracy. Combined strategies of our pseudopotential with softer pseudopotentials are also possible.

For instance, one could use a soft pseudopotential in structure-prediction workflows to pre-screen low-energy structures. To get a reliable energetic ranking, one could then recalculate a relatively small number of structures than with our highly accurate MPC pseudopotentials.

Our pseudopotential is however perfectly suitable as a production pseudopotential with all basis sets that allow for increasing the resolution in the region close to the nucleus such as Gaussian-type orbitals, numerical atomic orbitals,^49,55^ finite elements^56^ or multi-wavelets.^38,39^ In all these methods, the reduction of the number of electrons taken into consideration will speed up the calculations and allow to include in a very simple way relativistic effects in a non-relativistic calculation.

## Author contributions

Andris Gulans: test set design, LAPW and pseudopotential calculations, data analysis and management, writing – original draft, review and editing, visualization. Anders Brakestad: multiwavelet calculations. Stig Rune Jensen: multiwavelet code maintenance and availability. Moritz Gubler: multiwavelet code maintenance, data verification, review and editing. Luca Frediani: multiwavelet code maintenance and availability, review and editing. Stefan Goedecker: research concept, pseudopotential preparation, data analysis, writing – review and editing.

## Conflicts of interest

There are no conflicts to declare.

## Supplementary Material

CP-028-D6CP00528D-s001

CP-028-D6CP00528D-s002

## Data Availability

Inputs and outputs of calculations are available online in an open-data repository.^35,57^ See DOI: https://doi.org/10.17172/nomad.agf3-8cye. Supplementary information (SI) contains (i) a pdf document with a list of employed VASP PAW parametrizations (POTCARs) and diagrams with errors in atomization energies for additional PSPs, (ii) plain-text files with total and atomization energies obtained in all-electron and PSP calculations, (iii) a plain-text file with equilibrium bond lengths, (iv) the scalar-relativisitic multipole-conserving pseudopotentials given in the PSPPAR format. See DOI: https://doi.org/10.1039/d6cp00528d.
