# Supplementary material for: A synthetic pseudopotential benchmark for testing production pseudopotentials and constructing pseudopotentials with chemical accuracy
Source: Phys Chem Chem Phys. 2026 Sep 8;28(36):22013–27. doi: 10.1039/d6cp00528d (PMC13553045; doi:10.1039/d6cp00528d)
Supplement: Supplementary file 1 [file CP-028-D6CP00528D-s001.pdf]

Supplementary Information (SI) for Physical Chemistry Chemical Physics.
This journal is © the Owner Societies 2026

Supplementary information: A synthetic pseudopotential benchmark for testing production pseudopotentials and constructing pseudopotentials with chemical accuracy

Andris Gulans,[1,2] Anders Brakestad,[3,4] Stig Rune Jensen,[3,4] Moritz Gubler,[2,5] Luca Frediani,[3,4] and Stefan Goedecker[2]

[1] *Department of Physics, University of Latvia, Jelgavas iela 3, LV-1003 Riga, Latvia*
[2] *Department of Physics, Universitaet Basel, Klingelbergstrasse 82, CH-4056 Basel, Switzerland*
[3] *Hylleraas Centre for Quantum Molecular Sciences, UiT The Arctic University of Norway, 9037 Tromsø, Norway*
[4] *Department of Chemistry, UiT The Arctic University of Norway, 9037 Tromsø, Norway*
[5] *PSI Center for Scientific Computing, Theory and Data, Paul Scherrer Institute, 5232 Villigen PSI, Switzerland*

(Dated: September 8, 2026)

I. PSEUDOPOTENTIAL LIST

Tables I and II list the setups for the projector-augmented waves (PAW) used in VASP calculations in the present study.

Table I: PAW datasets employed in VASP benchmarks. The labels are consistent with the main text of the paper.

element	`VASP_std`	timestamp	`VASP_h`	timestamp	`VASP_GW`	timestamp
H	H	15Jun2001	H_h	06Feb2004	H_h_GW	21Apr2008
Li	Li_sv	23Jan2001	Li_sv	10Sep2004	Li_sv_GW	25Mar2010
Be	Be_sv	06Sep2000	Be_sv	06Sep2000	Be_sv_GW	31Mar2010
B	B	06Sep2000	B_h	06Feb2004	B_GW	28Sep2005
C	C	08Apr2002	C_h	06Feb2004	C_h_GW	23May2013
N	N	08Apr2002	N_h	06Feb2004	N_h_GW	03Jul2013
O	O	08Apr2002	O_h	06Feb2004	O_h_GW	22May2013
F	F	08Apr2002	F_h	31May2013	F_h_GW	20May2014
Na	Na_pv	05Jan2001	Na_sv	28Sep2000	Na_sv_GW	11May2015
Mg	Mg_pv	06Sep2000	Mg_sv	12Apr2007	Mg_sv_GW	20Apr2010
Al	Al	04Jan2001	Al	04Jan2001	Al_sv_GW	2Feb2008
Si	Si	05Jan2001	Si	05Jan2001	Si_sv_GW	29Sep2009
P	P	17Jan2003	P_h	08Apr2002	P_GW	19Mar2012
S	S	17Jan2003	S_h	08Apr2002	S_GW	19Mar2012
Cl	Cl	17Jan2003	Cl_h	21Jan2003	Cl_GW	19Mar2012
K	K_sv	06Sep2000	K_sv	06Sep2000	K_sv_GW	31Mar2010
Ca	Ca_sv	06Sep2000	Ca_sv	06Sep2000	Ca_sv_GW	31Mar2010
Sc	Sc_sv	07Sep2000	Sc_sv	07Sep2000	Sc_sv_GW	05Dec2013
Ti	Ti_pv	07Sep2000	Ti_sv	26Sep2005	Ti_sv_GW	05Dec2013
V	V_pv	07Sep2000	V_sv	02Aug2007	V_sv_GW	05Dec2013
Cr	Cr_pv	07Sep2000	Cr_sv	23Jul2007	Cr_sv_GW	05Dec2013
Mn	Mn_pv	07Sep2000	Mn_sv	23Jul2007	Mn_sv_GW	05Dec2013
Fe	Fe_pv	06Sep2000	Fe_sv	23Jul2007	Fe_sv_GW	05Dec2013
Co	Co	06Sep2000	Co_sv	23Jul2007	Co_sv_GW	05Dec2013
Ni	Ni_pv	06Sep2000	Ni_pv	06Sep2000	Ni_sv_GW	05Dec2013
Cu	Cu_pv	06Sep2000	Cu_pv	06Sep2000	Cu_sv_GW	05Dec2013
Zn	Zn	06Sep2000	Zn	06Sep2000	Zn_sv_GW	05Dec2013
Ga	Ga_d	06Sep2000	Ga_h	09Apr2002	Ga_sv_GW	05Dec2013
Ge	Ge_d	03Jul2007	Ge_h	09Apr2002	Ge_sv_GW	26Apr2015
As	As	06Sep2000	As_d	11Apr2003	As_sv_GW	05Dec2013
Se	Se	06Sep2000	Se	06Sep2000	Se_sv_GW	21Nov2013
Br	Br	06Sep2000	Br	06Sep2000	Br_sv_GW	21Nov2013
Rb	Rb_sv	06Sep2000	Rb_sv	06Sep2000	Rb_sv_GW	23Mar2010
Sr	Sr_sv	07Sep2000	Sr_sv	07Sep2000	Sr_sv_GW	23Mar2010
Y	Y_sv	06Sep2000	Y_sv	25May2007	Y_sv_GW	05Dec2013
Zr	Zr_sv	07Sep2000	Zr_sv	04Jan2005	Zr_sv_GW	05Dec2013
Nb	Nb_pv	08Apr2002	Nb_sv	25May2007	Nb_sv_GW	05Dec2013

Table II: PAW datasets employed in VASP benchmarks - continued.

element	std	timestamp	hard	timestamp	GW-ready	timestamp
Mo	Mo_pv	08Apr2002	Mo_sv	02Feb2006	Mo_sv_GW	05Dec2013
Tc	Tc_pv	06Sep2000	Tc_sv	23Mar2010	Tc_sv_GW	05Dec2013
Ru	Ru_pv	06Sep2000	Ru_sv	28Jan2005	Ru_sv_GW	05Dec2013
Rh	Rh_pv	06Sep2000	Rh_pv	25Jan2005	Rh_sv_GW	05Dec2013
Pd	Pd	05Jan2001	Pd_pv	28Jan2005	Pd_sv_GW	05Dec2013
Ag	Ag	06Sep2000	Ag_pv	09Dec2005	Ag_sv_GW	05Dec2013
Cd	Cd	06Sep2000	Cd	06Sep2000	Cd_sv_GW	16Apr2014
In	In_d	06Sep2000	In_d	06Sep2000	In_sv_GW	22Nov2013
Sn	Sn_d	06Sep2000	Sn_d	06Sep2000	Sn_sv_GW	22Nov2013
Sb	Sb	06Sep2000	Sb	06Sep2000	Sb_sv_GW	22Nov2013
Te	Te	08Apr2002	Te	08Apr2002	Te_sv_GW	22Nov2013
I	I	08Apr2002	I	08Apr2002	I_sv_GW	22Nov2013
Cs	Cs_sv	08Apr2002	Cs_sv	08Apr2002	Cs_sv_GW	23Mar2010
Ba	Ba_sv	06Sep2000	Ba_sv	06Sep2000	Ba_sv_GW	23Mar2010
Lu	Lu_3	06Sep2000	Lu	23Dec2003	–	–
Hf	Hf_pv	06Sep2000	Hf_sv	10Jan2008	Hf_sv_GW	16Jan2015
Ta	Ta_pv	07Sep2000	Ta_pv	07Sep2000	Ta_sv_GW	23Mar2010
W	W_pv	06Sep2000	W_sv	04Sep2015	W_sv_GW	23Mar2010
Re	Re_pv	06Sep2000	Re_pv	06Sep2000	Re_sv_GW	23Mar2010
Os	Os_pv	20Jan2003	Os_pv	20Jan2003	Os_sv_GW	23Mar2010
Ir	Ir	06Sep2000	Ir	06Sep2000	Ir_sv_GW	23Mar2010
Pt	Pt	05Jan2001	Pt_pv	12Dec2005	Pt_sv_GW	23Mar2010
Au	Au	06Sep2000	Au	04Oct2007	Au_sv_GW	13Sep2013
Hg	Hg	06Sep2000	Hg	06Sep2000	Hg_sv_GW	16Apr2014
Tl	Tl_d	06Sep2000	Tl_d	06Sep2000	Tl_sv_GW	16Apr2014
Pb	Pb_d	06Sep2000	Pb_d	06Sep2000	Pb_sv_GW	04Apr201z
Bi	Bi	08Apr2002	Bi_d	06Sep2000	Bi_sv_GW	14Apr2014
Po	Po	10Feb2004	Po_d	25May2007	Po_sv_GW	14Apr2014

II. PSEUDOPOTENTIAL ANALYSIS DATA

Figures 2-9 summarize performance of the pseudopotentials (PSP) for which the error summaries were not presented in the main text of the paper. The color-coding and the layout are explained in the main text of the paper.

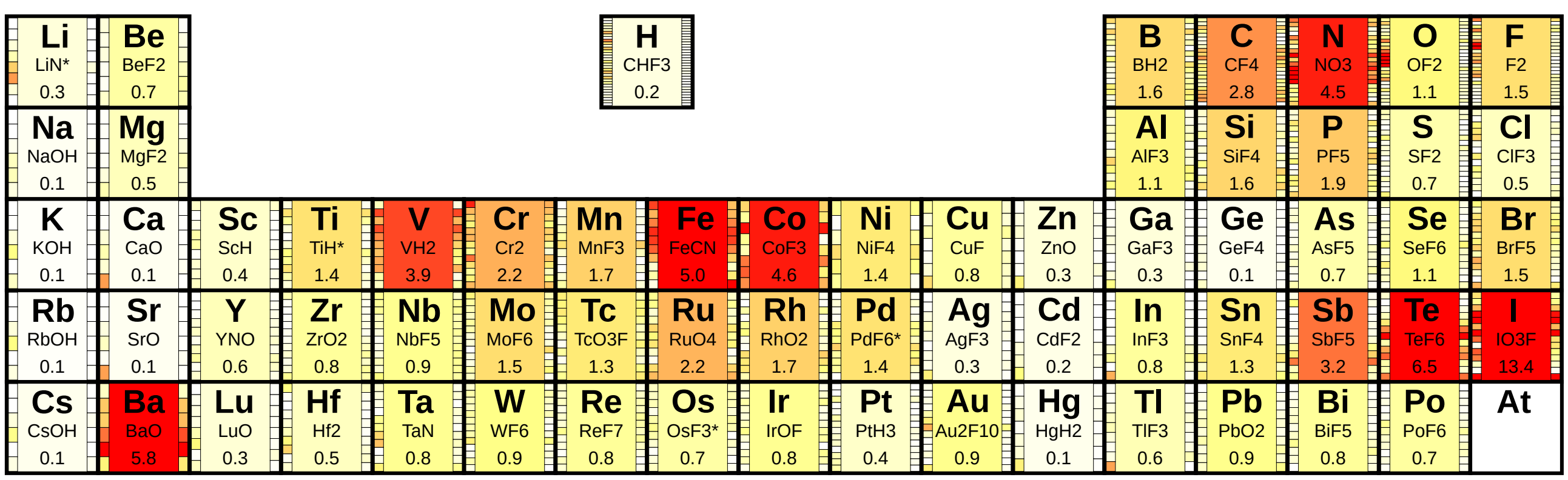

Figure 1: Errors in atomization energies due to the ONCV (version 0.4) PSPs.

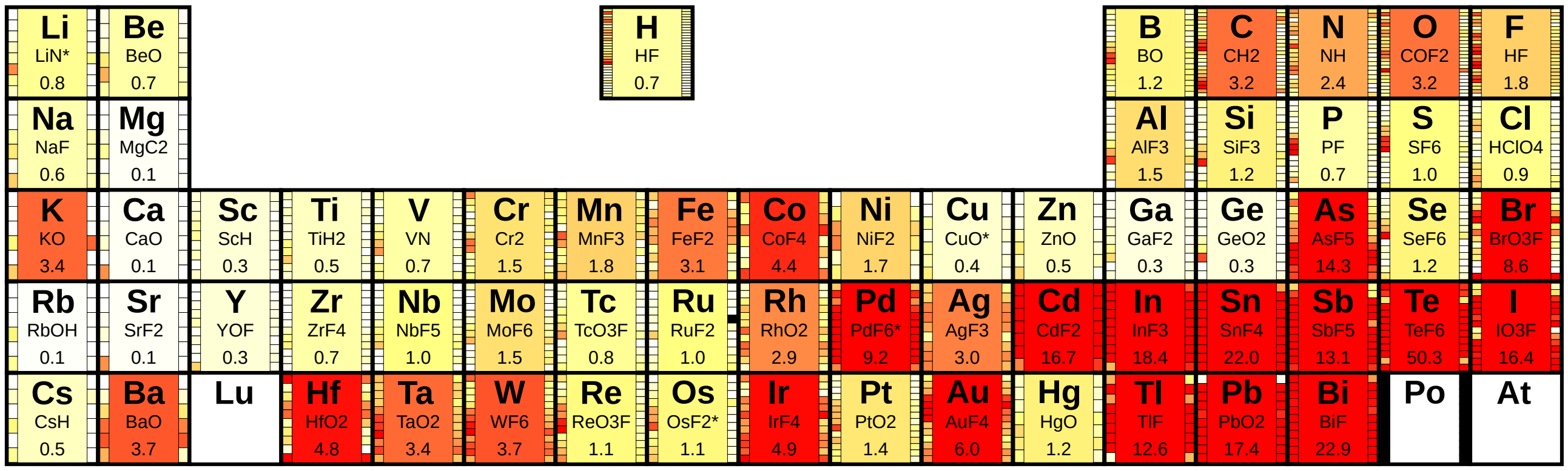

Figure 2: Errors in atomization energies due to the GBRV PSPs.

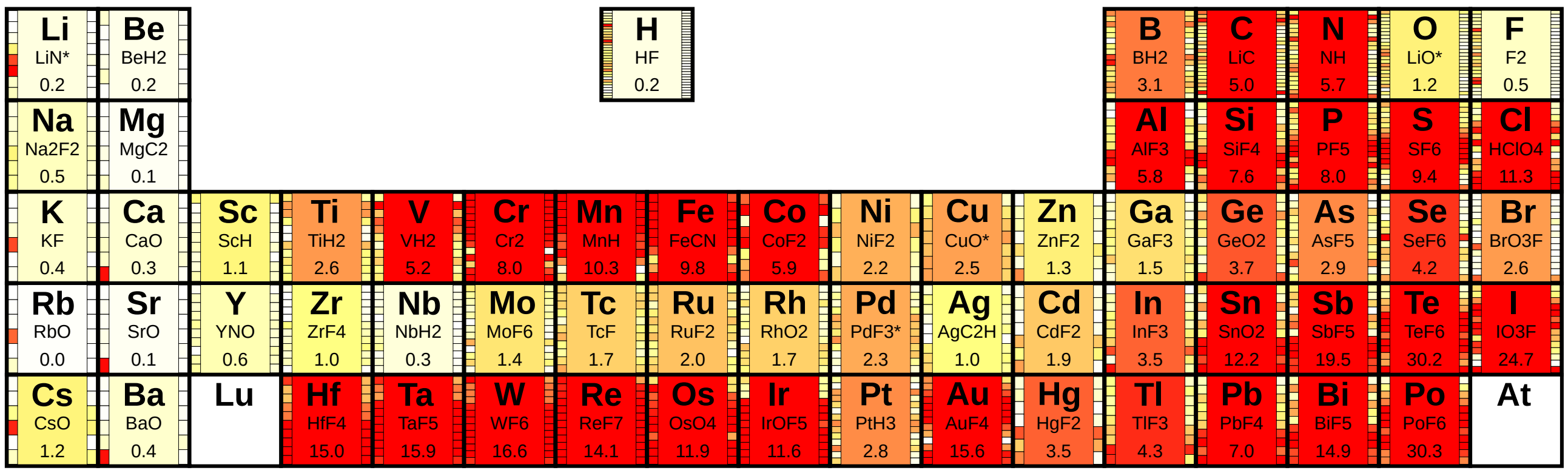

Figure 3: Errors in atomization energies due to the HGH PSPs.

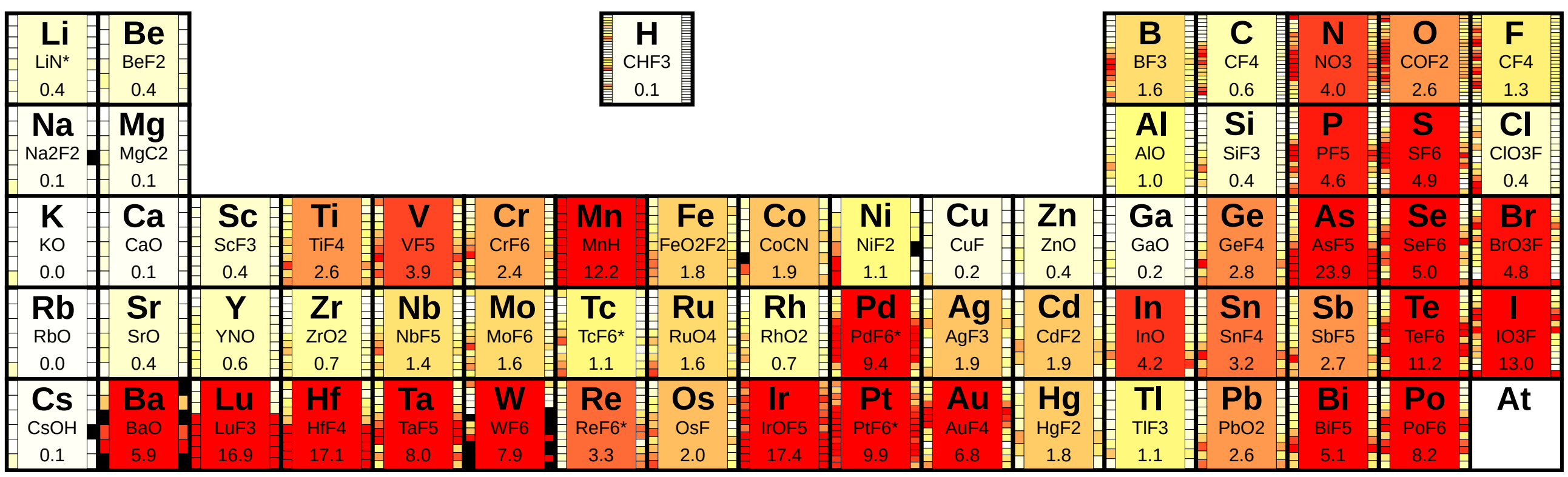

Figure 4: Errors in atomization energies due to the kjpaw (pslibrary) PSPs.

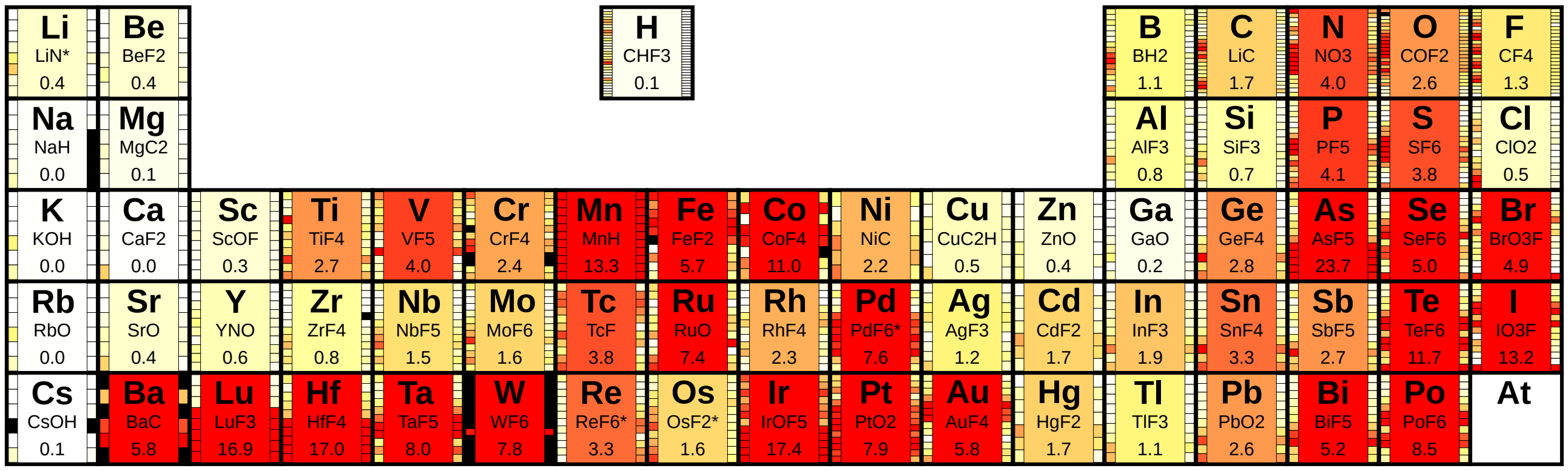

Figure 5: Errors in atomization energies due to the rrkjus (pslibrary) PSPs.

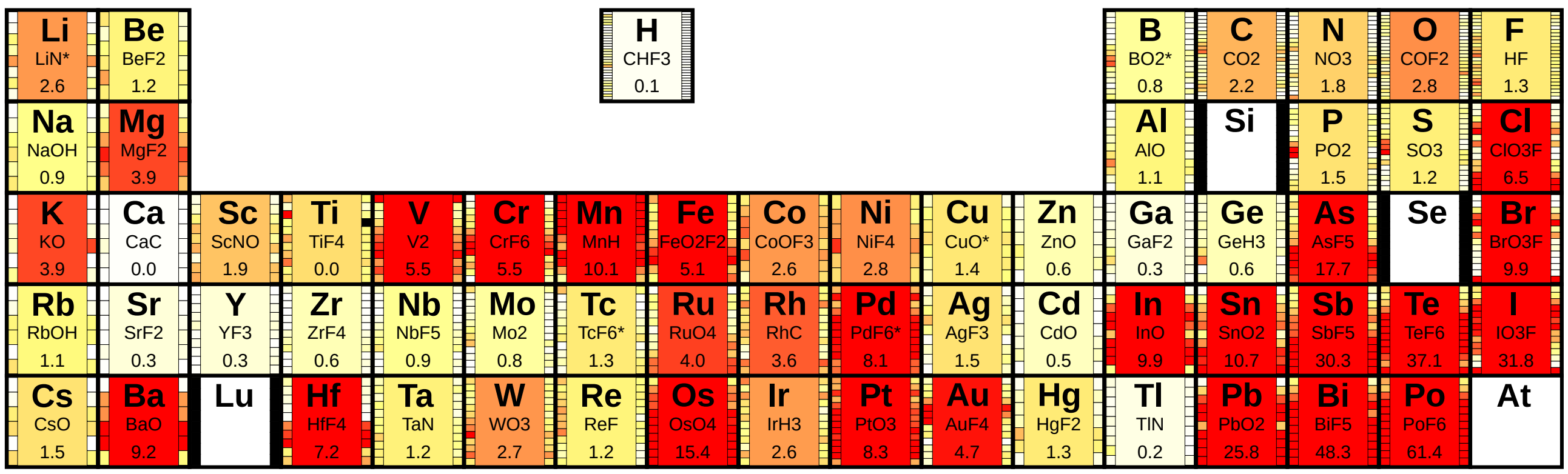

Figure 6: Errors in atomization energies due to the JTH (v1.0) PSPs.

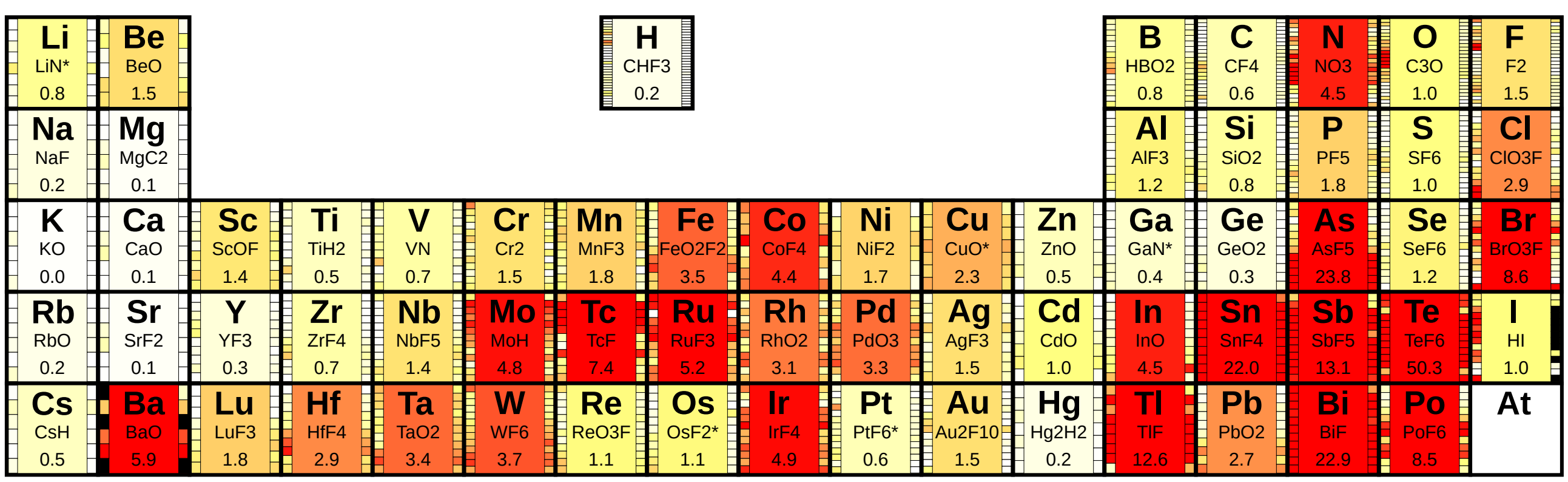

Figure 7: Errors in atomization energies due to the SSSP (precision, version 1.0) PSPs.

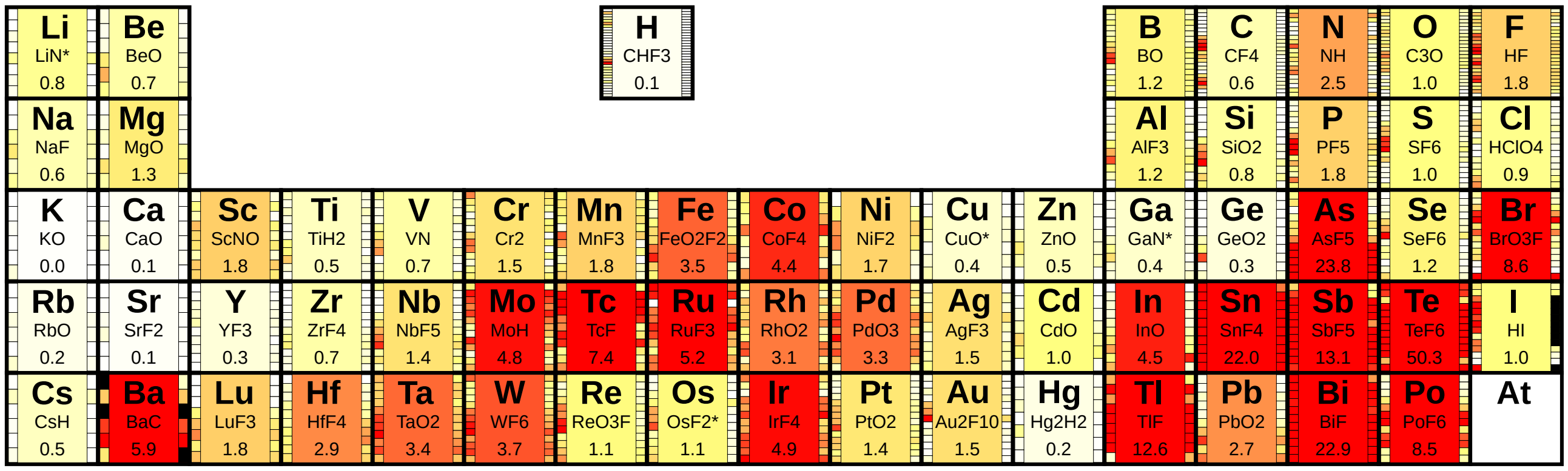

Figure 8: Errors in atomization energies due to the SSSP (efficiency, version 1.0) PSPs.

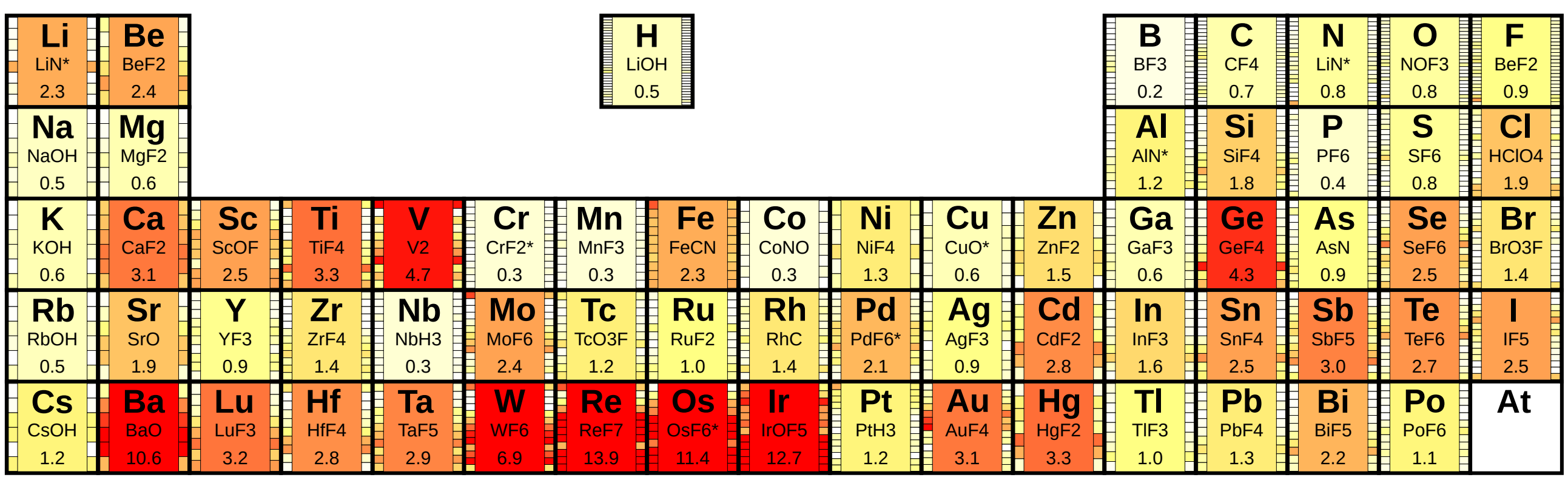

Figure 9: Errors in atomization energies due to the VASP PAWs labeled as *hard* or corresponding to the smallest core for a given element. The color-coding and the layout are explained in the main text of the paper.
